# Perioperative Factor Xa Inhibitor Discontinuation for Patients Undergoing Procedures With Minimal or Low Bleeding Risk

**DOI:** 10.1001/jamanetworkopen.2024.58742

**Published:** 2025-02-07

**Authors:** So-Ryoung Lee, Kyung-Yeon Lee, Jong-Sung Park, Young Soo Lee, Yong Seog Oh, Sang-Jin Han, June Namgung, Ji Hyun Lee, Woo-Hyun Lim, Min Soo Ahn, Soonil Kwon, Hyo-Jeong Ahn, Seil Oh, Gregory Y. H. Lip, Eue-Keun Choi

**Affiliations:** 1 Department of Internal Medicine, Seoul National University Hospital, Seoul, Republic of Korea; 2 Department of Internal Medicine, Seoul National University College of Medicine, Seoul, Republic of Korea; 3 Department of Cardiology, Dong-A University Hospital, Busan, Republic of Korea; 4 Department of Cardiology, Daegu Catholic University Medical Center, Daegu, Republic of Korea; 5 Department of Cardiology, The Catholic University of Korea, St Mary’s Hospital, Seoul, Republic of Korea; 6 Department of Internal Medicine, College of Medicine, Hallym University, Anyang, Republic of Korea; 7 Division of Cardiology, Department of Internal Medicine, Inje University Ilsan Paik Hospital, Ilsan, Republic of Korea; 8 Department of Internal Medicine, Seoul National University Bundang Hospital, Seoul, Republic of Korea; 9 Department of Internal Medicine, SMG-SNU Boramae Medical Center, Seoul, Republic of Korea; 10 Department of Internal Medicine, Yonsei University Wonju College of Medicine, Wonju, Republic of Korea; 11 Liverpool Centre for Cardiovascular Science, University of Liverpool, Liverpool, United Kingdom; 12 Danish Center for Health Services Research, Department of Clinical Medicine, Aalborg University, Aalborg, Denmark; 13 Liverpool John Moores University and Liverpool Heart & Chest Hospital, Liverpool, United Kingdom

## Abstract

**Importance:**

Discontinuation of oral anticoagulant treatment is common in clinical practice due to concerns about bleeding, even for procedures with minimal to low bleeding risk.

**Objective:**

To explore whether perioperative discontinuation of factor Xa inhibitors is associated with major bleeding and thromboembolic events in patients with atrial fibrillation (AF) undergoing procedures with minimal to low bleeding risk.

**Design, Setting, and Participants:**

This prospective, multicenter, single-arm cohort study conducted in Korea included patients with AF who planned to undergo a procedure with minimal to low bleeding risk between September 25, 2020, and April 5, 2024.

**Exposure:**

The PERIXa (Perioperative Factor Xa Inhibitor Discontinuation in Patients With Atrial Fibrillation Undergoing Minimal to Low Bleed Risk Procedures) protocol recommending giving the last dose of factor Xa inhibitor (ie, apixaban, edoxaban, or rivaroxaban) 24 hours before the procedure (ie, endoscopy, dental procedure, or ocular surgery) and restarting treatment with the inhibitor the next day.

**Main Outcomes and Measures:**

The primary outcome was major bleeding, and the secondary outcome included a composite of thromboembolic events 30 days after the index procedure with minimal to low bleeding risk.

**Results:**

In total, 1902 patients were included in the modified intention-to-treat analysis set encompassing all patients who underwent the intended procedure (mean [SD] age, 70.4 [8.8] years; 1135 [59.7%] male; mean [SD] CHA_2_DS_2_-VASc [congestive heart failure, hypertension, age 75 years or older, diabetes, stroke, vascular disease, age 65-74 years, and female sex; range, 0-9, with higher scores indicating higher risk of stroke] score, 2.8 [1.3]; mean [SD] HAS-BLED [hypertension, kidney or liver disease, stroke history, prior bleeding, unstable international normalized ratio, age >65 years, and drug or alcohol use; range, 0-9, with higher scores indicating higher risk of bleeding] score, 1.6 [0.7]). Among them, 921 (48.4%) were receiving apixaban, 616 (32.4%) were receiving edoxaban, and 365 (19.2%) were receiving rivaroxaban. Of the total procedures, 948 (49.8%) were endoscopy, 820 (43.1%) were dental procedures, and 120 (6.3%) were ocular surgery. The 30-day event rate of major bleeding was 0.1% (n = 2), and there were no composite thromboembolic events. The results were consistent in the per-protocol analysis, and no differences were observed by procedure category or factor Xa inhibitor type.

**Conclusions and Relevance:**

In this cohort study, patients with AF receiving a factor Xa inhibitor and undergoing a procedure with minimal to low bleeding risk had low rates of major bleeding and thromboembolism when following the standardized PERIXa protocol for perioperative management of oral anticoagulant treatment, suggesting that this may be a safe and reasonable option for this patient population.

## Key Points

**Question:**

Is the use of PERIXa, the standardized protocol for perioperative factor Xa inhibitor discontinuation and resumption, associated with rates of major bleeding and thromboembolism among patients with atrial fibrillation (AF) undergoing procedures with minimal to low bleeding risk?

**Findings:**

In this cohort study involving 1902 patients with AF who were receiving factor Xa inhibitors and planned to undergo procedures with minimal to low bleeding risk, including endoscopy, dental procedures, or ocular surgery, the 30-day postprocedural event rates were 0.1% for major bleeding and 0% for thromboembolism.

**Meaning:**

The findings of this cohort study suggest that the standardized perioperative anticoagulation strategy in the PERIXa protocol may be a safe and reasonable option for patients with AF undergoing procedures with minimal to low bleeding risk.

## Teaser

This cohort study investigates the safety and effectiveness of the standardized PERIXa protocol for perioperative discontinuation of factor Xa inhibitors among patients with atrial fibrillation undergoing procedures with minimal or low bleeding risk.

## Introduction

Oral anticoagulants (OACs) are crucial in preventing and treating thromboembolism in patients with atrial fibrillation (AF).^1,2^ Multiple trials and clinical practice studies have consistently demonstrated the efficacy and safety of direct oral anticoagulants (DOACs).^3^ These studies demonstrate that DOACs offer a similar or lower risk of stroke and significantly reduce intracranial hemorrhage compared with warfarin.^3^ Consequently, DOACs have largely supplanted warfarin and addressed the previous underuse of OACs.^4,5^

Approximately 1 in 4 patients receiving anticoagulants requires temporary discontinuation of their medication for a planned procedure within 2 years.^6^ Annually, 15% to 20% of patients undergoing anticoagulant therapy require a procedure or surgery.^6,7,8,9,10^ With the increasing number of patients receiving DOAC therapy, temporary interruptions for elective procedures or surgery have become more common. Specific management of DOACs during the perioperative period has been suggested based on the type of DOAC and the bleeding risk of the procedure.^11^ However, there is a lack of robust evidence supporting current recommendations for perioperative DOAC management, especially for procedures deemed to have minimal to low bleeding risk by the International Society on Thrombosis and Haemostasis (ISTH)^12^ (overlapping with minor bleeding-risk intervention according to the European Heart Rhythm Association [EHRA] classification^11^), such as gastrointestinal tract endoscopy, dental procedures, and ocular surgery.

Although bleeding risk stratification and recommendations for these procedures have been somewhat established in the field of cardiology,^8,11,12^ guidelines from different specialties offer varying recommendations on managing DOACs for these procedures.^13^ For instance, cardiology and gastroenterology societies differ in their definitions of procedures with minimal to low bleeding risk and advice on whether to continue or interrupt DOAC therapy.^14,15,16,17^ Similarly, dental and ophthalmology guidelines lack consistent definitions and recommendations for procedures with minimal to low bleeding risk.^18,19,20,21^ These inconsistencies underscore the need for a practical, standardized protocol for managing DOACs during procedures with minimal to low bleeding risk.

Therefore, we designed the PERIXa study (Perioperative Factor Xa Inhibitor Discontinuation in Patients With Atrial Fibrillation Undergoing Minimal to Low Bleed Risk Procedures) to explore the association of short-term perioperative discontinuation of factor Xa inhibitors with rates of major bleeding and thromboembolism among patients with AF undergoing procedures with minimal to low bleeding risk.

## Methods

### Study Design and Oversight

The PERIXa study was a multicenter, prospective, single-arm cohort study conducted at 29 sites in South Korea. The study rationale, detailed study design, and protocol are described elsewhere.^13^ Details regarding the participating centers and investigators are provided in Supplement 1. This study adhered to the ethical principles outlined in the Declaration of Helsinki (2013 revision).^22^ The institutional review board of each participating center approved the PERIXa study, and all participants provided written informed consent. This report followed the Strengthening the Reporting of Observational Studies in Epidemiology (STROBE) reporting guideline for cohort studies.

The PERIXa study focused on patients with AF who were being treated with factor Xa inhibitors and needed to interrupt anticoagulation therapy for elective procedures with minimal to low bleeding risk. Patients were allocated to 1 of 3 cohorts based on the type of procedure (endoscopy, dental, or ocular) and also classified into 3 groups according to the specific factor Xa inhibitor used (ie, apixaban, edoxaban, or rivaroxaban).^11^

The EHRA offers a pragmatic guideline suggesting DOAC discontinuation 18 to 24 hours before an intervention and resumption 6 hours afterward, based on the DOAC half-life or elimination time.^11^ However, this recommendation lacks robust, large-scale clinical evidence. In the PERIXa study, we proposed and evaluated a more user-friendly protocol based on the EHRA guideline for DOAC discontinuation before and resumption after procedures with minimal to low bleeding risk defined by the ISTH Guidance Statement for the risk stratification for procedural bleeding risk.^12^ The procedures included in the PERIXa study align with the categories of minimal and low bleeding risk defined by the ISTH Guidance Statement and overlap with the minor-risk and low-risk interventions suggested by EHRA.^11^ These classifications are summarized in eAppendix 7 in Supplement 2. Our primary outcome was focused on major bleeding as defined by the ISTH.^23^

### Study Population and Procedure

Between September 25, 2020, and April 5, 2024, eligible participants included adults with AF receiving a factor Xa inhibitor (ie, apixaban, edoxaban, and rivaroxaban) who were scheduled to undergo procedures with minimal to low bleeding risk, including endoscopy, dental procedures, and ocular surgery. Full eligibility criteria are provided in eAppendix 1 in Supplement 2.

Patients who were referred by the operator to cardiologists for preprocedural consultations regarding DOAC discontinuation before procedures with minimal to low bleeding risk were screened by the cardiologist. eAppendix 2 in Supplement 2 provides the list of procedures with minimal to low bleeding risk. After providing written informed consent, eligible patients were enrolled. Once enrolled, patients were informed of the preprocedure and postprocedure drug discontinuation protocols, and operators of procedures with minimal to low bleeding risk were also informed of study enrollment and the factor Xa inhibitor discontinuation protocol in a protocolized document.

The PERIXa discontinuation protocol is provided in eFigure 1A in Supplement 2. The PERIXa protocol was largely based on the EHRA protocol, with modifications to make it more user-friendly and clearer for each factor Xa inhibitor application. For apixaban, taken twice daily, the protocol was to discontinue the evening dose a day before the procedure and the morning dose on the procedure day. If the procedure was performed in the morning, the evening dose could be restarted after hemostasis was achieved. The usual protocol was to resume the regular schedule the next morning. For once-daily edoxaban and rivaroxaban, the protocol was to discontinue on the morning of the procedure and resume the next morning.^11,13^

For patients who underwent the planned procedure, we examined the actual procedure details, the duration of drug discontinuation, and the clinical outcomes immediately after the procedure, as well as at 7 and 30 days after the procedure (eFigure 1B in Supplement 2). Additionally, the operator who performed the procedure was asked to provide details about the actual procedure and the level of bleeding and hemostasis observed during the operation. The survey questions are available in eAppendix 3 in Supplement 2.

### Outcomes and Definitions

The primary outcome was the occurrence of major bleeding, as defined by the ISTH, within 30 days following the index procedure.^23^ The secondary outcomes were a 30-day composite of thromboembolic events, including stroke, transient ischemic attack, systemic embolism, and myocardial infarction. Also, clinically relevant nonmajor bleeding (CRNMB), minor bleeding, and all bleeding (a composite of major bleeding, CRNMB, and minor bleeding) were collected for secondary outcomes.^23^ Additionally, individual components of thromboembolic events and death from any cause were collected, and a composite of all thromboembolic events and death from any cause were additional secondary outcomes. The list and definitions of outcomes are provided in eAppendices 4 and 5 in Supplement 2.

### Statistical Analysis

The study used a superiority design. The sample size was calculated based on a primary outcome of a 30-day major bleeding event, defined by ISTH criteria, with a significance level of 2-sided *P* < .05 and 80% power.^24,25,26,27^ Details regarding the sample size estimation and any amendments to it are provided in eAppendix 6 in Supplement 2. Based on these assumptions, a sample size of 2303 subjects was required, which was increased to 2500 to account for an anticipated 8% dropout rate.

All analyses followed the modified intention-to-treat principle, encompassing all patients who underwent the intended procedure with minimal to low bleeding risk. Sensitivity analyses were carried out in the per-protocol population, including patients with periprocedural DOAC interruptions according to the predefined protocol. Outcomes were analyzed and reported for the total population, as well as stratified by procedure type, type of DOAC, and dosing regimen (once or twice daily). Continuous variables are expressed as mean (SD) or median with IQR and were analyzed using the *t* test, 1-way analysis of variance, or Kruskal-Wallis test, as appropriate. Categorical variables are reported as frequencies, and percentages are compared using the χ^2^ test or Fisher exact test. R, version 4.2.3 (R Project for Statistical Computing), was used for all statistical analysis. Statistical significance was determined with a 2-sided *P* < .05. The study, registered at ClinicalTrials.gov, has been completed.^28^

## Results

### Study Patients

A total of 2500 patients were enrolled across 29 participating centers. After excluding 598 patients, primarily because the planned procedure with minimal to low bleeding risk was not performed, 1902 patients were included in the modified intention-to-treat primary analyses (Figure). The mean (SD) age was 70.4 (8.8) years, with 612 patients (32.2%) 75 years of age or older, and 1135 male (59.7%) and 767 female (40.3%) patients (Table 1). In the modified intention-to-treat analysis set, the mean (SD) CHA_2_DS_2_-VASc (congestive heart failure, hypertension, age 75 years or older, diabetes, stroke, vascular disease, age 65-74 years, and female sex; range, 0-9, with higher scores indicating higher risk of stroke) score was 2.8 (1.3), with 1053 of 1886 patients (55.8%) having a score of 3 or higher. The mean (SD) HAS-BLED (hypertension, kidney or liver disease, stroke history, prior bleeding, unstable international normalized ratio, age >65 years, and drug or alcohol use; range, 0-9, with higher scores indicating higher risk of bleeding) score was 1.6 (0.7), with 165 of 1884 patients (8.8%) having a score of 3 or higher. Among 1902 patients, 921 (48.4%) were receiving apixaban, 616 (32.4%) were receiving edoxaban, and 365 (19.2%) were receiving rivaroxaban.

**Figure.**
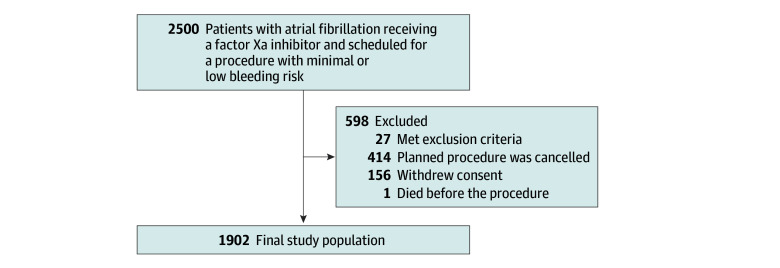
Study Flow

**Table 1.** Baseline Characteristics According to the Procedures in the Modified Intention-to-Treat Analysis Set

Characteristic	Patients, No. (%)					*P* value
	Overall (N = 1902)	Endoscopy (n = 948)	Dental procedure (n = 820)	Ocular surgery (n = 120)	Other procedures (n = 14)	
Age, median (IQR), y	70.5 (65.0-76.0)	69.5 (65.0-75.0)	72.0 (65.0-78.5)	75.0 (69.0-81.0)	70.5 (64.0-73.0)	<.001
<65	411 (21.6)	234 (24.7)	163 (19.9)	10 (8.3)	4 (28.6)	
65-74	879 (46.2)	469 (49.5)	353 (43.0)	49 (40.8)	8 (57.1)	
≥75	612 (32.2)	245 (25.8)	304 (37.1)	61 (50.8)	2 (14.3)	
Sex, No. (%)						
Female	767 (40.3)	362 (38.2)	344 (42.0)	56 (46.7)	5 (35.7)	.18
Male	1135 (59.7)	586 (61.8)	476 (58.0)	64 (53.3)	9 (64.3)	
CHA_2_DS_2_-VASc, median (IQR)^a^	3.0 (2.0-4.0)	2.0 (2.0-3.0)	3.0 (2.0-4.0)	3.0 (3.0-4.0)	2.0 (1.0-3.0)	<.001
CHA_2_DS_2_-VASc <3	833 (44.2)	474 (50.3)	321 (39.7)	28 (23.3)	10 (71.4)	<.001
CHA_2_DS_2_-VASc ≥3	1053 (55.8)	469 (49.7)	488 (60.3)	92 (76.7)	4 (28.6)	
HAS-BLED, median (IQR)^b^	2.0 (1.0-2.0)	2.0 (1.0-2.0)	2.0 (1.0-2.0)	2.0 (1.0-2.0)	1.5 (1.0-2.0)	.02
HAS-BLED <3	1719 (91.2)	860 (91.4)	732 (90.5)	113 (94.2)	14 (100)	.36
HAS-BLED ≥3	165 (8.8)	81 (8.6)	77 (9.5)	7 (5.8)	0	
Comorbidities						
Hypertension	1300 (68.3)	642 (67.7)	560 (68.3)	88 (73.3)	10 (71.4)	.66
Diabetes	499 (26.2)	227 (23.9)	223 (27.2)	47 (39.2)	2 (14.3)	.002
CHF	257 (13.5)	112 (11.8)	121 (14.8)	24 (20.0)	0	.02
CKD	106 (5.6)	44 (4.6)	51 (6.2)	11 (9.2)	0	.11
Dialysis	9 (0.5)	2 (0.2)	6 (0.7)	1 (0.8)	0	.40
Chronic liver disease	28 (1.5)	14 (1.5)	12 (1.5)	2 (1.7)	0	.97
Stroke, TIA, or TE	162 (8.5)	72 (7.6)	84 (10.2)	6 (5.0)	0	.06
Previous DCC	329 (17.3)	172 (18.1)	138 (16.8)	15 (12.5)	4 (28.6)	.29
Previous RFCA	417 (21.9)	243 (25.6)	154 (18.8)	15 (12.5)	5 (35.7)	<.001
Previous ACS	45 (2.4)	12 (1.3)	28 (3.4)	5 (4.2)	0	.01
Previous PCI	82 (4.3)	31 (3.3)	46 (5.6)	5 (4.2)	0	.09
Medications						
Apixaban	921 (48.4)	469 (49.5)	391 (47.7)	53 (44.2)	8 (57.1)	.60
5 mg Twice daily	679 (35.7)	368 (38.8)	270 (32.9)	35 (29.2)	6 (42.9)	.04
2.5 mg Twice daily	242 (12.7)	101 (10.7)	121 (14.8)	18 (15.0)	2 (14.3)	
Edoxaban	616 (32.4)	321 (33.9)	253 (30.9)	38 (31.7)	4 (28.6)	.59
60 mg Once daily	312 (16.4)	168 (17.7)	125 (15.2)	15 (12.5)	4 (28.6)	.05
30 mg Once daily	292 (15.4)	152 (16.0)	118 (14.4)	22 (18.3)	0	
15 mg once daily	12 (0.6)	1 (0.1)	10 (1.2)	1 (0.8)	0	
Rivaroxaban	365 (19.2)	158 (16.7)	176 (21.5)	29 (24.2)	2 (14.3)	.03
20 mg once daily	136 (7.2)	61 (6.4)	67 (8.2)	8 (6.7)	0	<.001
15 mg once daily	222 (11.7)	97 (10.2)	103 (12.6)	21 (17.5)	1 (7.1)	
10 mg once daily	7 (0.4)	0	6 (0.7)	0	1 (7.1)	
Class Ic AAD	683 (35.9)	366 (38.6)	287 (35.0)	24 (20.0)	6 (42.9)	.001
Class III AAD	316 (16.6)	172 (18.1)	124 (15.1)	18 (15.0)	2 (14.3)	.36
β Blocker	971 (51.1)	477 (50.3)	426 (52.0)	60 (50.0)	8 (57.1)	.87
CCB	1254 (65.9)	626 (66.0)	547 (66.7)	76 (63.3)	5 (35.7)	.10
Digoxin	94 (4.9)	37 (3.9)	50 (6.1)	7 (5.8)	0	.14
ACEI	48 (2.5)	19 (2.0)	25 (3.0)	4 (3.3)	0	.45
ARB	745 (39.2)	366 (38.6)	327 (39.9)	49 (40.8)	3 (21.4)	.52
Diuretic	424 (22.3)	186 (19.6)	201 (24.5)	36 (30.0)	1 (7.1)	.007
Statin	917 (48.2)	465 (49.1)	397 (48.4)	52 (43.3)	3 (21.4)	.14
NSAID	10 (0.5)	3 (0.3)	6 (0.7)	1 (0.8)	0	.63
PPI	293 (15.4)	173 (18.2)	108 (13.2)	12 (10.0)	0	.003
H2 blocker	41 (2.2)	24 (2.5)	16 (2.0)	1 (0.8)	0	.55
Laboratory findings						
Platelet, median (IQR), ×10^3^/μL	201.0 (168.5-239.0)	203.0 (170.0-241.0)	200.5 (167.0-236.5)	196.5 (158.5-250.0)	217.0 (185.0-221.5)	.84
PT, median (IQR), INR	1.1 (1.0-1.3)	1.1 (1.0-1.2)	1.1 (1.0-1.3)	1.1 (1.0-1.4)	1.1 (1.1-1.2)	.39
APTT, median (IQR), s	32.8 (29.1-37.0)	32.0 (29.1-36.9)	33.7 (28.9-37.4)	33.6 (29.4-37.4)	30.8 (29.1-37.8)	.49
Creatinine, median (IQR), mg/dL	0.9 (0.8-1.0)	0.9 (0.8-1.0)	0.9 (0.8-1.1)	0.9 (0.8-1.1)	0.9 (0.8-0.9)	.06
eGFR, MDRD, median (IQR), mL/min/1.73 m^2^	75.9 (64.9-87.0)	77.6 (66.8-88.6)	74.6 (63.6-85.3)	69.3 (54.3-82.7)	82.7 (75.7-89.6)	<.001
AST, median (IQR), IU/L	23.0 (20.0-29.0)	24.0 (20.0-29.0)	23.0 (19.0-29.0)	22.0 (19.0-25.0)	21.5 (20.0-25.0)	.04
ALT, median (IQR), IU/L	19.0 (15.0-27.0)	20.0 (15.0-27.0)	19.0 (14.0-27.0)	17.0 (12.0-21.5)	18.5 (12.0-23.0)	.003
LA volume, median (IQR), mL	63.9 (47.5-91.0)	64.5 (47.3-91.0)	62.0 (47.6-87.0)	77.0 (54.0-119.9)	54.0 (39.0-69.0)	.33
LA volume index, median (IQR), mL/m^2^	44.2 (34.9-58.0)	43.5 (35.4-56.0)	44.0 (33.6-60.3)	51.4 (41.6-66.5)	32.8 (20.7-44.8)	.15
LV ejection fraction, median (IQR), %	60.0 (56.0-64.0)	60.0 (56.0-65.0)	59.0 (56.0-63.0)	60.5 (56.0-63.9)	66.0 (62.5-70.4)	.01
BMI, median (IQR)	24.8 (22.7-26.9)	24.6 (22.7-26.7)	24.9 (22.7-27.1)	24.8 (22.8-27.0)	26.0 (22.7-27.9)	.61
Smoking status						
Unknown	830 (43.6)	457 (48.2)	311 (37.9)	55 (45.8)	7 (50.0)	.006
Never	889 (46.7)	406 (42.8)	425 (51.8)	52 (43.3)	6 (42.9)	
Former (quit >2 mo ago)	104 (5.5)	47 (5.0)	51 (6.2)	5 (4.2)	1 (7.1)	
Current	79 (4.2)	38 (4.0)	33 (4.0)	8 (6.7)	0	
Alcohol consumption						
No	827 (43.5)	382 (40.3)	387 (47.2)	52 (43.3)	6 (42.9)	.02
Social	114 (6.0)	52 (5.5)	58 (7.1)	4 (3.3)	0	
Yes	183 (9.6)	93 (9.8)	79 (9.6)	11 (9.2)	0	
Unknown	778 (40.9)	421 (44.4)	296 (36.1)	53 (44.2)	8 (57.1)	

Abbreviations: AAD, antiarrhythmic drug; ACEI, angiotensin-converting enzyme inhibitor; ACS, acute coronary syndrome; ALT, alanine aminotransferase; APTT, activated partial thromboplastin time; ARB, angiotensin receptor blocker; AST, aspartate aminotransferase; BMI, body mass index (calculated as weight in kilograms divided by height in meters squared); CCB, calcium channel blocker; CHA_2_DS_2_-VASc, congestive heart failure, hypertension, age 75 years or older, diabetes, stroke, vascular disease, age 65-74 years, and female sex; CHF, congestive heart failure; CKD, chronic kidney disease; DCC, direct current cardioversion; eGFR, estimated glomerular filtration rate; H2, histamine 2 receptor; HAS-BLED, hypertension, kidney or liver disease, stroke history, prior bleeding, unstable international normalized ratio, age >65 years, and drug or alcohol use; INR, international normalized ratio; LA, left atrium; LV, left ventricle; MDRD, modification of diet in renal disease; NSAID, nonsteroidal anti-inflammatory drug; PCI, percutaneous coronary intervention; PPI, proton-pump inhibitor; PT, prothrombin time; RFCA, radiofrequency catheter ablation; TE, thromboembolism; TIA, transient ischemic attack.

SI conversion factors: To convert platelet count to ×10^9^/L, multiply by 1.0; creatinine levels to micromoles per liter, multiply 88.4; AST and ALT to microkatals per liter, multiply by 0.0167.

^a^ CHA_2_DS_2_-VASc scores range from 0 to 9, with higher scores indicating higher risk of stroke.

^b^ HAS-BLED scores range from 0 to 9, with higher scores indicating higher risk of bleeding.

Of the total procedures with minimal to low bleeding risk, 948 (49.8%) were endoscopy, 820 (43.1%) were dental procedures, and 120 (6.3%) were ocular surgeries (eFigure 2 in Supplement 2); 948 (49.8%) were low bleeding risk (endoscopy) and the remaining 954 (50.2%) procedures were minimal bleeding risk according to the ISTH classification. A detailed classification within the procedure categories is presented in eTable 1 in Supplement 2. In a brief look at procedures, patients who underwent ocular surgery were oldest, with the highest percentage of patients aged 75 years or older (61 [50.8%]) and the highest CHA_2_DS_2_-VASc scores. Baseline characteristics by DOAC type and DOAC regimen are detailed in eTable 2 in Supplement 2. No significant differences were observed in CHA_2_DS_2_-VASc or HAS-BLED scores among the groups for each DOAC, whether administered once or twice daily.

### Study Outcomes

In the modified intention-to-treat analysis set, the 30-day postprocedural event rate of major bleeding was 0.1% (2 patients), and the 30-day postprocedural event rate for the composite of thromboembolic events was 0 (Table 2). The rates of major bleeding across the procedure categories (endoscopy, 0; dental 2 [0.2]%; ocular surgery 0) and the rates of a composite of thromboembolic events were similar (0 for all procedures), as were the rates of major bleeding by DOAC type (0 for apixaban, 2 [0.3%] for edoxaban, and 0 for rivaroxaban) and DOAC regimen (0 for twice daily and 2 [0.2%] for once daily) and a composite of thromboembolic events by DOAC type (0 for all) and DOAC regimen (0 for once or twice daily) (Table 2 and Table 3).

**Table 2.** Clinical Outcomes According to the Procedures in the Modified Intention-to-Treat Analysis Set

Outcome	Outcome, No. (%)					*P* value	*P* value^a^
	Overall (N = 1902)	Endoscopy (n = 948)	Dental procedure (n = 820)	Ocular surgery (n = 120)	Other procedure (n = 14)		
Primary							
Major bleeding	2 (0.1)	0	2 (0.2)	0	0	.45	.27
Secondary							
Composite of thromboembolic events^b^	0	0	0	0	0	NA	NA
Bleeding							
CRNMB	8 (0.4)	1 (0.1)	7 (0.9)	0	0	.09	.04
Minor	43 (2.3)	7 (0.7)	34 (4.2)	0	2 (16.7)	<.001	<.001
All	50 (2.6)	7 (0.7)	41 (5.0)	0	2 (14.3)	<.001	<.001
Thromboembolic event							
Stroke	0	0	0	0	0	NA	NA
Transient ischemic attack	0	0	0	0	0	NA	NA
Systemic embolism	0	0	0	0	0	NA	NA
Myocardial infarction	0	0	0	0	0	NA	NA
Other	0	0	0	0	0	NA	NA
Death from any cause	0	0	0	0	0	NA	NA
Composite of thromboembolic event and all-cause death	0	0	0	0	0	NA	NA

Abbreviations: CRNMB, clinically relevant nonmajor bleeding; NA, not applicable.

^a^ *P* value calculated excluding the procedure category other.

^b^ Including stroke, transient ischemic attack, systemic embolism, and myocardial infarction.

**Table 3.** Clinical Outcomes by DOAC Type and Regimen in the Modified Intention-to-Treat Analysis Set

Outcome	Outcome by DOAC type, No. (%)			*P* value	Outcome by DOAC regimen, No. (%)		*P* value
	Apixaban (n = 921)	Edoxaban (n = 616)	Rivaroxaban (n = 365)		Twice daily (n = 921)	Once daily (n = 981)	
Primary outcome							
Major bleeding	0	2 (0.3)	0	.12	0	2 (0.2)	.51
Secondary outcome							
Composite of thromboembolic events^a^	0	0	0	NA	0	0	NA
Bleeding							
CRNMB	7 (0.8)	0	1 (0.3)	.07	7 (0.8)	1 (0.1)	.06
Minor	21 (2.3)	11 (1.8)	11 (3.1)	.45	21 (2.3)	22 (2.3)	1
All	26 (2.8)	12 (1.9)	12 (3.3)	.39	26 (2.8)	24 (2.4)	.71
Thromboembolic event							
Stroke	0	0	0	NA	0	0	NA
Transient ischemic attack	0	0	0	NA	0	0	NA
Systemic embolism	0	0	0	NA	0	0	NA
Myocardial infarction	0	0	0	NA	0	0	NA
Other	0	0	0	NA	0	0	NA
Death from any cause	0	0	0	NA	0	0	NA
Composite of thromboembolic events and all-cause death	0	0	0	NA	0	0	NA

Abbreviations: CRNMB, clinically relevant nonmajor bleeding; DOAC, direct oral anticoagulant; NA, not applicable.

^a^ Including stroke, transient ischemic attack, systemic embolism, and myocardial infarction.

Regarding secondary outcomes other than the composite of thromboembolic events, the 30-day postprocedural event rates were 0.4% (8 patients) for CRNMB, 2.3% (43 patients) for minor bleeding, and 2.6% (50 patients) for all bleeding. The CRNMB event rate was numerically higher in dental procedures than those for endoscopy or ocular surgery but without a statistically significant difference. Minor bleeding was also higher in the dental procedure category, as was all bleeding except in other categories where the type of procedure was heterogeneous.

There were no differences in the event rates of primary and secondary outcomes among DOAC types (apixaban, edoxaban, or rivaroxaban) and no between DOAC regimens (once or twice daily). The events of each outcome of the visit are summarized in eTable 3 in Supplement 2.

### Results of Questionnaire Acquired From Operators

Within the modified intention-to-treat analysis set of PERIXa-enrolled and scheduled procedures, the in-procedure survey response rate for bleeding and bleeding control was 60.9% of operators who conducted the procedures (n = 1158) (Table 4). Among the responses, 260 operators (22.5%) indicated no concerns about bleeding during and immediately after the procedure, while 760 (65.6%) found the bleeding comparable to that expected in general patients not receiving anticoagulation. Additionally, 133 operators (11.5%) noted more bleeding during the procedure compared with patients not receiving anticoagulation, although it remained controllable. A small percentage, 0.3% (n = 3, all in the dental procedure group), reported requiring additional measures to manage bleeding during the procedure, and 0.1% (n = 1, in the endoscopy group) needed further intervention to control delayed bleeding after the procedure. For dental procedures, operators were significantly more likely to report that bleeding was greater than that expected in the general population of patients without anticoagulation (101 [19.0%] for dental procedures, 27 [5.0%] for endoscopy, and 3 [4.1%] for ocular surgery (*P* < .001). There were no significant differences between groups in operator-perceived intraprocedural bleeding, immediate postprocedural bleeding, or bleeding control based on DOAC type (eTable 4 in Supplement 2) or DOAC regimen (eTable 5 in Supplement 2).

**Table 4.** Postprocedural Questionnaire Responses From the Operators in the Modified Intention-to-Treat Analysis Set

Survey response	Respondents, No. (%)^a^^,^^b^				
	Overall (n = 1902)	Endoscopy (n = 948)	Dental procedure (n = 820)	Ocular surgery (n = 120)	Other procedure (n = 14)
Completed survey	1158 (60.9)	543 (57.3)	532 (64.9)	74 (61.7)	9 (64.3)
No bleeding at all	260 (22.5)	184 (33.9)	50 (9.4)	24 (32.4)	2 (22.2)
Bleeding similar to the expected level in a general patient not receiving anticoagulation	760 (65.6)	331 (61.0)	377 (70.9)	47 (63.5)	5 (55.6)
More bleeding during or immediately after the procedure than in a general patient not receiving anticoagulation, but controllable during the procedure	133 (11.5)	27 (5.0)	101 (19.0)	3 (4.1)	2 (22.2)
More bleeding during or immediately after the procedure than in a general patient not receiving anticoagulation, requiring special measures for hemostasis	3 (0.3)	0	3 (0.6)	0	0
Postprocedure or surgery reintervention or additional endoscopy due to delayed bleeding	1 (0.1)	1 (0.2)	0	0	0
Other bleeding	1 (0.1)	0	1 (0.2)	0	0

^a^ Percentages calculated based on the number of respondents.

^b^ *P* < .001.

### Sensitivity Analysis

The adherence rate for the factor Xa inhibitor treatment interruption and resumption protocol was 84.9% (n = 1615). The most common deviation from the perioperative OAC discontinuation and resumption protocol was the discontinuation of the agent earlier than indicated in the preprocedure protocol (166 of 1902 [8.7%]) (eTable 6 in Supplement 2). The baseline characteristics of study participants in the per-protocol analysis set are presented in eTable 7 in Supplement 2.

In the per-protocol analysis set, the major bleeding event rate was 0.1%, and the rate of the composite of thromboembolic events was 0%, neither of which was significantly different from the modified intention-to-treat analysis set (eTable 8 in Supplement 2). Survey responses among operators who performed the procedure were also not significantly different from the modified intention-to-treat analysis set (eTable 9 in Supplement 2).

## Discussion

In this prospective, multicenter cohort study of patients with AF who were receiving factor Xa inhibitor anticoagulation treatment and undergoing procedures with minimal to low bleeding risk, the PERIXa protocol for factor Xa inhibitor interruption and resumption was associated with a low 30-day major bleeding event rate (0.1%) and no thromboembolic events (eFigure 3 in Supplement 2). Among 1902 patients included in the modified intention-to-treat analysis set, there were 2 (0.1%) major bleeding events, 8 (0.4%) CRNMB, 43 (2.3%) minor bleeding, and 50 (2.6%) all bleeding events that occurred.

Dental procedures had the highest incidence of bleeding events among the 3 categories of procedures with minimal to low bleeding risk. Bleeding event rates were not significantly different across various types of DOACs (ie, apixaban, edoxaban, or rivaroxaban) or dosing regimens (once or twice daily). Periprocedural protocol adherence was 84.9%. The per-protocol analysis corroborated the main findings.

The EHRA practical guidelines advise against discontinuing OACs for scheduled procedures with minor bleeding risk, recommending that they be performed 12 to 24 hours after the last DOAC dose.^11^ However, a more practical clinical approach may involve administering the final DOAC dose 18 to 24 hours before the procedure and resuming it 6 hours after.^11^ An inconsistency exists between this general guideline and specific practical guidelines that suggest single-dose discontinuation based on the DOAC type, such as apixaban or dabigatran. Furthermore, there is a lack of prospective evidence supporting this recommendation. The American College of Chest Physicians clinical practice guideline for perioperative management of antithrombotic therapy published in 2022^8^ recommended continuing anticoagulation treatment for procedures with minimal bleeding risk with no or only minimal interruption (ie, on the day of the procedure). For procedures with low to moderate bleeding risk, they advise pausing 1 day before the operation and resuming DOACs at least 24 hours after the procedure.^8^ However, this guideline did not offer unified recommendations for endoscopy, dental, or ocular procedures, categorized as interventions with minor bleeding risk in the EHRA guideline.^11^ While there is limited research supporting recommendations for procedures with minimal bleeding risk, our PERIXa study provides substantial observational evidence supporting the perioperative discontinuation of factor Xa inhibitors in a large prospective cohort.

Smaller studies suggest reduced bleeding risk when anticoagulation is discontinued rather than continued without significantly increasing the risk of stroke or systemic embolism.^25,29,30,31^ Subanalyses of pivotal randomized clinical trials—comparing the efficacy and safety of 4 DOACs to warfarin—provided insights into periprocedural management strategies for DOACs, reaching similar conclusions.^10,32,33,34^ These subanalyses of pivotal randomized clinical trials had limitations: few patients underwent scheduled procedures with minimal to low bleeding risk, and there were no standardized protocols for DOAC discontinuation. Nevertheless, the findings indicated that temporarily stopping DOAC administration during the periprocedural period may lower the risk of major bleeding without substantially increasing thromboembolic events.^10,32,33,34^

Prospective studies have evaluated periprocedural management of DOACs, including a meta-analysis comparing DOACs with warfarin in elective procedures under a noninterruption or short interruption strategy.^35^ This analysis demonstrated a 38% lower risk of major bleeding with DOAC treatment under a noninterruption strategy, with no differences observed under an interrupted strategy while maintaining comparable efficacy in stroke prevention and overall mortality.^35^ However, no prospective studies have focused on procedures with minimal to low bleeding risk. The PAUSE (Perioperative Anticoagulant Use for Surgery Evaluation) trial was the largest multicenter, multinational clinical study using a 1-arm design with a well-defined periprocedural DOAC management protocol for 3007 patients with AF undergoing procedures with high and low to moderate bleeding risk.^36^ The PAUSE trial found endoscopic procedures comprised 20.9% of total procedures, with ocular and dental procedures at 0.7% and 0.3%, respectively.^24^ Other studies^37,38^ and a registry^29^ reported that endoscopic procedures comprised 19.9% to 34.9% of the total procedures, while ocular or dental procedures accounted for only 3.9% to 13.4%. Although the PAUSE trial^24^ found very low rates of major bleeding and thromboembolism (major bleeding rates of 0.9%-1.9% and stroke or thromboembolism rates of 0.2%-0.6% at 30 days after the procedure), with other studies reporting similar event rates,^10,25,29,30,31,32,33,34^ there was a small proportion of procedures with minimal bleeding risk included. The PERIXa study aligns with and expands on the findings of the PAUSE study in periprocedural DOAC management. The present study used data from a large multicenter study with a well-defined interruption protocol for factor Xa inhibitors in patients with AF undergoing procedures with minimal to low bleeding risk.

### Strengths and Limitations

A strength of the PERIXa study lies in its prospective enrollment of 1902 patients undergoing planned procedures with minimal to low bleeding risk, along with its inclusion of valuable edoxaban periprocedural data (n = 616 [32.4%]), which is limited in this research field. This study demonstrated a simplified DOAC interruption protocol before and after these interventions, making communicating with patients and health care professionals easier. This protocol resulted in a very low major bleeding event rate and no thromboembolic events at 30 days. A recent review^39^ proposes a slightly shorter, near noninterruption protocol than the PERIXa study. Their protocol suggests omitting twice daily DOACs only on the morning of the procedure and delaying once daily DOACs slightly beyond their usual ingestion time. However, this recommendation lacks support from evidence encompassing a wide range of large-scale procedures or 3 categories of procedures with minimal to low bleeding risk.^40,41,42,43^ Moreover, the absence of a defined discontinuation protocol can raise patient and health care professionals’ concerns about postprocedure hemostasis difficulties, potentially leading to unnecessary and prolonged DOAC discontinuation.

Another novel aspect of this study is our survey of operators who performed procedures with minimal to low bleeding risk asking about bleeding and hemostasis during and after the procedure. The questionnaire return rate was 60.9%, which suggests potential selection bias. Notably, in 88.1% of patients with no bleeding issues, DOAC discontinuation following the PERIXa protocol was perceived by operators to be comparable to the general population without anticoagulation treatment. This finding was similarly reflected in the per-protocol analysis set at 88.9% (eTable 9 in Supplement 2).

Our study has several limitations. First, we planned to enroll 2500 patients to account for the dropout rate, expecting a final analysis of 2303 patients. However, due to a higher than anticipated dropout rate—including patients scheduled for procedures with minimal to low bleeding risk who did not receive them—we analyzed fewer patients than initially planned. Nevertheless, 1902 patients included in the modified intention-to-treat analysis represent the largest enrollment in any published prospective multicenter study examining the safety of DOAC interruption for procedures with minimal to low bleeding risk. Second, our study observed relatively low event rates compared with prior research. The PAUSE trial, for example, which included about 20% procedures with minimal to low bleeding risk, reported 30-day major bleeding rates of 0.9% to 1.9% and thromboembolic risks of 0.2% to 0.6% across its entire cohort.^24^ That trial had higher mean CHA_2_DS_2_-VASc (3.5) and modified HAS-BLED (2.0) scores than the PERIXa study cohort (2.8 and 1.6, respectively). Given that our study focused exclusively on procedures with minimal to low bleeding risk, our observed event rates—0.1% major bleeding, 0.4% CRNMB, 2.3% minor bleeding, and 2.6% overall bleeding, with no thromboembolic events—are considered relevant in comparison to previous studies. However, in the endoscopy category, unlike dental procedures and ocular surgery, there may be underreporting of nonsignificant bleeding events. Third, while most procedures in our study were classified as minimal to low bleeding risk, a small percentage (14 [0.7%]) of procedures were ambiguous in classification or required additional intraprocedural treatment and were classified as other procedures, thereby increasing the anticipated bleeding risk (eTable 1 in Supplement 2). For instance, 67 (7%) of 948 endoscopy cases involved unplanned therapeutic interventions, such as endoscopic mucosal resection or dissection. This finding reflects a plausible clinical scenario that may arise in routine practice. Although these unplanned higher-risk interventions were not associated with any major bleeding events in our study, the clinical implications of such unexpected interventions within the PERIXa protocol across different categories warrant further investigation. Fourth, this study applied OAC interruption and resumption according to a predefined protocol, observed periprocedural clinical events, and could not evaluate clinical outcomes following an uninterrupted DOAC strategy for procedures with minimal bleeding risk. Fifth, surveys completed by operators who performed procedures formed the basis for evaluating study outcomes rather than direct verification by researchers. This approach may introduce bias into the reported outcomes due to operators’ subjective assessments. Lastly, despite the multicenter design, this study was conducted exclusively in Korea. This limits the study population to individuals of Asian race and ethnicity, thereby restricting the generalizability of the findings to other racial and ethnic groups.

## Conclusions

In this prospective, multicenter cohort study, patients with AF who were receiving factor Xa inhibitors and undergoing procedures with minimal to low bleeding risk had low rates of major bleeding and thromboembolism when following the standardized PERIXa protocol for perioperative management. Implementation studies are needed to assess the feasibility of integrating the PERIXa protocol into routine clinical practice, including potential barriers and facilitators to adoption.

## Notes

Supplement 1. **Nonauthor Collaborators.** The PERIXa Investigators

Supplement 2. **eAppendix 1.** Inclusion and exclusion criteria **eAppendix 2.** The list of minimal and low bleed risk intervention **eAppendix 3.** The contents of the post-procedural questionnaire **eAppendix 4.** Full list of primary and secondary outcomes **eAppendix 5.** Definitions of study outcomes **eAppendix 6.** Sample size estimation **eAppendix 7.** Risk stratification for procedural bleed risk of the procedures included in the PERIXa study from the ISTH and EHRA **eTable 1.** Procedure characteristics in the modified intention-to-treat analysis set **eTable 2.** Baseline characteristics according to the DOAC types and DOAC regimens in the modified intention-to-treat analysis set **eTable 3.** Clinical outcomes at each visit according to the procedures in the modified intention-to-treat analysis set **eTable 4.** Post-procedural questionnaire from the operators according to the DOAC types in the modified intention-to-treat analysis set **eTable 5.** Post-procedural questionnaire from the operators according to the DOAC regimens in the modified intention-to-treat analysis set **eTable 6.** The reason for protocol violation in the modified intention-to-treat analysis set **eTable 7.** Baseline characteristics of participants in the per-protocol analysis set **eTable 8.** Clinical Outcomes of participants who followed protocol according to the procedure in the per-protocol analysis set **eTable 9.** Post-procedural questionnaire from the operators for participants in the per-protocol analysis set **eFigure 1.** Perioperative direct oral anticoagulant management protocol for minimal and low bleed risk intervention and PERIXa study design **eFigure 2.** Procedure characteristics **eFigure 3.** Central Illustration: a simplified protocol for the discontinuation and resumption of factor Xa inhibitor periprocedurally in patients with atrial fibrillation undergoing minimal to low bleed risk procedures

Supplement 3.
Data Sharing Statement
