# Supplementary material for: Perioperative Factor Xa Inhibitor Discontinuation for Patients Undergoing Procedures With Minimal or Low Bleeding Risk
Source: JAMA Netw Open. 2025 Feb 7;8(2):e2458742. doi: 10.1001/jamanetworkopen.2024.58742 (PMC11806392; doi:10.1001/jamanetworkopen.2024.58742)
Supplement: Supplement 1. — Nonauthor Collaborators. The PERIXa Investigators [file jamanetwopen-e2458742-s001.pdf]

Supplemental Online Content: Nonauthor Collaborators
*First name, last name, and suffix (if applicable) are required and will appear in PubMed.

| *Group Name(s): The PERIXa investigators | | | | | | | |
|---|---|---|---|---|---|---|---|
| ***First Name and Middle Initial(s)** | ***Last Name** | ***Suffix (eg, Jr, III)** | Academic Degrees | Institution | Location (city, state/province, country) | Role or Contribution, eg, chair, principal investigator | Group (if more than 1 Group listed in the byline) and/or Subgroup (eg, Steering Committee) |
| Sung-Won | Jang | | MD, PhD | The Catholic University of Korea, Eunpyeong St. Mary's Hospital | Seoul, Korea | Site PI | |
| Jong-Il | Choi | | MD, PhD | Korea University Anam Hospital | Seoul, Korea | Site PI | |
| Jung Ho | Heo | | MD, PhD | Kosin University Gospel Hospital | Busan, Korea | Site PI | |
| Junbeom | Park | | MD, PhD | Ewha Womans University Medical C | Seoul, Korea | Site PI | |
| Moo-Nyun | Jin | | MD, PhD | Ewha Womans University Medical C | Seoul, Korea | Co-Investigator | |
| Ki-Woon | Kang | | MD, PhD | Chung-Ang University Hospital | Seoul, Korea | Site PI | |
| Sun Hwa | Kim | | MD | Presbyterian Medical Center | Jeonju, Korea | Site PI | |
| Namsik | Yoon | | MD, PhD | Chonnam National University Hospit | Gwangju, Korea | Site PI | |
| Yong-Soo | Baek | | MD, PhD | Inha University Hospital | Incheon, Korea | Site PI | |
| Sung Ho | Lee | | MD, PhD | Kangbuk Samsung Hospital | Seoul, Korea | Site PI | |
| Tae-Hoon | Kim | | MD | Severance Hospital | Seoul, Korea | Site PI | |
| Hee Tae | Yu | | MD, PhD | Severance Hospital | Seoul, Korea | Co-Investigator | |
| Seung-Young | Roh | | MD, PhD | Korea University Guro Hospital | Seoul, Korea | Site PI | |
| Kwang Jin | Chun | | MD, PhD | Kangwon National Unversity Hospita | Chuncheon, Korea | Site PI | |

*First name, last name, and suffix (if applicable) are required and will appear in PubMed.

| ***First Name and Middle Initial(s)** | ***Last Name** | ***Suffix (eg, Jr, III)** | Academic Degrees | Institution | Location (city, state/province, country) | Role or Contribution, eg, chair, principal investigator | Group (if more than 1 Group listed in the byline) and/or Subgroup (eg, Steering Committee) |
|---|---|---|---|---|---|---|---|
| Moo-Nyun | Jin | | MD, PhD | Inje University Sanggye Paik Hospita | Seoul, Korea | Site PI | |
| Ki-Byung | Nam | | MD, PhD | Asan Medical Center | Seoul, Korea | Site PI | |
| Seongwook | Han | | MD, PhD | Keimyung University Dongsan Medi | Daegu, Korea | Site PI | |
| Kwang-No | Lee | | MD, PhD | Ajou University Medical Center | Suwon, Korea | Site PI | |
| Je-Wook | Park | | MD | Yongin Severance Hospital | Yongin, Korea | Site PI | |
| Jae-Sun | Uhm | | MD, PhD | Yongin Severance Hospital | Yongin, Korea | Co-Investigator | |
| Jung Hoon | Sung | | MD, PhD | Cha Bundang Medical Center | Bundang, Korea | Site PI | |
| Young Keun | On | | MD, PhD | Samsung Medical Center | Seoul, Korea | Site PI | |
| Sung-Soo | Lee | | MD | Soonchunhyang University Hospital | Seoul, Korea | Site PI | |
