# Supplementary material for: Perioperative Factor Xa Inhibitor Discontinuation for Patients Undergoing Procedures With Minimal or Low Bleeding Risk
Source: JAMA Netw Open. 2025 Feb 7;8(2):e2458742. doi: 10.1001/jamanetworkopen.2024.58742 (PMC11806392; doi:10.1001/jamanetworkopen.2024.58742)
Supplement: Supplement 2. — eAppendix 1. Inclusion and exclusion criteria eAppendix 2. The list of minimal and low bleed risk intervention eAppendix 3. The contents of the post-procedural questionnaire eAppendix 4. Full list of primary and secondary outcomes eAppendix 5. Definitions of study outcomes eAppendix 6. Sample size estimation eAppendix 7. Risk stratification for procedural bleed risk of the procedures included in the PERIXa study from the ISTH and EHRA eTable 1. Procedure characteristics in the modified intention-to-treat analysis set eTable 2. Baseline characteristics according to the DOAC types and DOAC regimens in the modified intention-to-treat analysis set eTable 3. Clinical outcomes at each visit according to the procedures in the modified intention-to-treat analysis set eTable 4. Post-procedural questionnaire from the operators according to the DOAC types in the modified intention-to-treat analysis set eTable 5. Post-procedural questionnaire from the operators according to the DOAC regimens in the modified intention-to-treat analysis set eTable 6. The reason for protocol violation in the modified intention-to-treat analysis set eTable 7. Baseline characteristics of participants in the per-protocol analysis set eTable 8. Clinical Outcomes of participants who followed protocol according to the procedure in the per-protocol analysis set eTable 9. Post-procedural questionnaire from the operators for participants in the per-protocol analysis set eFigure 1. Perioperative direct oral anticoagulant management protocol for minimal and low bleed risk intervention and PERIXa study design eFigure 2. Procedure characteristics eFigure 3. Central Illustration: a simplified protocol for the discontinuation and resumption of factor Xa inhibitor periprocedurally in patients with atrial fibrillation undergoing minimal to low bleed risk procedures [file jamanetwopen-e2458742-s002.pdf]

Supplementary Online Content

Lee SR, Lee KY, Park JS, et al. Perioperative factor Xa inhibitor discontinuation for patients undergoing procedures with minimal or low bleeding risk. *JAMA Netw Open*. 2024;8(2):e2458739. doi:10.1001/jamanetworkopen.2024.58739

eAppendix 1. Inclusion and exclusion criteria

eAppendix 2. The list of minimal and low bleed risk intervention

eAppendix 3. The contents of the post-procedural questionnaire

eAppendix 4. Full list of primary and secondary outcomes

eAppendix 5. Definitions of study outcomes

eAppendix 6. Sample size estimation

eAppendix 7. Risk stratification for procedural bleed risk of the procedures included in the PERIXa study from the ISTH and EHRA

eTable 1. Procedure characteristics in the modified intention-to-treat analysis set

eTable 2. Baseline characteristics according to the DOAC types and DOAC regimens in the modified intention-to-treat analysis set

eTable 3. Clinical outcomes at each visit according to the procedures in the modified intention-to-treat analysis set

eTable 4. Post-procedural questionnaire from the operators according to the DOAC types in the modified intention-to-treat analysis set

eTable 5. Post-procedural questionnaire from the operators according to the DOAC regimens in the modified intention-to-treat analysis set

eTable 6. The reason for protocol violation in the modified intention-to-treat analysis set

eTable 7. Baseline characteristics of participants in the per-protocol analysis set

eTable 8. Clinical Outcomes of participants who followed protocol according to the procedure in the per-protocol analysis set

eTable 9. Post-procedural questionnaire from the operators for participants in the per-protocol analysis set

© 2025 Lee SR et al. *JAMA Network Open*.

eFigure 1. Perioperative direct oral anticoagulant management protocol for minimal and low bleed risk intervention and PERIXa study design

eFigure 2. Procedure characteristics

eFigure 3. Central Illustration: a simplified protocol for the discontinuation and resumption of factor Xa inhibitor periprocedurally in patients with atrial fibrillation undergoing minimal to low bleed risk procedures

eReferences.

This supplementary material has been provided by the authors to give readers additional information about their work.

© 2025 Lee SR et al. *JAMA Network Open*.

eAppendix 1. Inclusion and exclusion criteria

**Inclusion Criteria**
1. Age 20 years or older<br>2. Patients with non-valvular atrial fibrillation taking direct oral anticoagulants (apixaban, edoxaban, and rivaroxaban)<br>3. Patients scheduled for procedures defined as minor bleeding risk procedures<br>4. Willing and able to provide informed written consent
**Exclusion Criteria**
1. Pregnant women or other vulnerable subjects<br>2. Patient taking once a daily agent (rivaroxaban or edoxaban) regularly in the afternoon schedule<br>3. Patient with severe psychiatric or cognitive impairment who are expected to have poor adherence to this study<br>4. Patient with contraindications for rivaroxaban, apixaban, or edoxaban according to the local marketing authorization/summary of medicinal products characteristics<br>5. Patient with a diagnosis of moderate or severe mitral stenosis or who have undergone prosthetic valve replacement surgery<br>6. Patient with indications for OACs other than AF (ie, pulmonary artery thrombus or deep vein thrombus)<br>7. Patient who are scheduled for another procedure or surgery with a minor bleeding-risk or greater bleeding-risk within 30 days of an index minor bleeding-risk procedure or surgery<br>8. Patient taking OACs or antiplatelet agent(s) other than rivaroxaban, apixaban, or edoxaban<br>9. Patient with major bleeding, systemic embolism, or those who experienced stroke in the past 12 months<br>10. Patient with a planned therapeutic endoscopic procedure

© 2025 Lee SR et al. *JAMA Network Open*.

eAppendix 2. The list of minimal and low bleed risk intervention

Minimal and Low Bleed Risk Interventions	Endoscopy	• Diagnostic gastroduodenoscopic procedures ± biopsy[a]<br>• Diagnostic colonoscopic procedures ± biopsy[a]
	Dental procedures	• 1–3 teeth extraction<br>• Periodontal surgery<br>• Incisions and Drainage<br>• Dental implants
	Ocular surgery	• Cataract<br>• Glaucoma surgery

Notes: [a]Diagnostic esophagogastroduodenoscopy, colonoscopy, sigmoidoscopy, and biopsy, except for polypectomy, endoscopic mucosal resection, endoscopic submucosal dissection, and endoscopic variceal ligation, endoscopic hemostasis, pneumatic or bougie dilation, endoscopic ultrasound-guided fine-needle aspiration, percutaneous endoscopic gastrostomy, percutaneous endoscopic jejunostomy, and therapeutic balloon-assisted enteroscopy (including esophageal, enteral, and colonic stenting).

© 2025 Lee SR et al. *JAMA Network Open*.

eAppendix 3. The contents of the post-procedural questionnaire

What was the effect of anticoagulant discontinuation on bleeding during or after the procedure/surgery for the patient?

A. None at all

B. Similar to the expected level in a general patient not on anticoagulation

C. More bleeding during or immediately after the procedure than in a general patient not on anticoagulation, but controllable during the procedure (e.g., hemoclip, argon plasma coagulation, band ligation) to the level of minor bleeding

D. More bleeding during or immediately after the procedure than in a general patient not on anticoagulation, requiring special measures for hemostasis (e.g., vascular embolization, emergency surgery) or transfusion to the level of major bleeding

E. Post-procedure/surgery re-intervention or additional endoscopy due to delayed bleeding

F. Other

The questionnaire employed in this study was developed through extensive discussions with investigators and experts in relevant fields, including dentistry, gastroenterology, and ophthalmology.

© 2025 Lee SR et al. *JAMA Network Open*.

eAppendix 4. Full list of primary and secondary outcomes

Primary outcome	30-day major bleeding event according to the ISTH definition(1)
Secondary outcomes - Bleedings	(1) 30-day clinically relevant nonmajor bleeding<br>(2) 30-day minor bleeding<br>(3) 30-day all bleeding (a composite of major bleeding, clinically relevant nonmajor bleeding, and minor bleeding)
Secondary outcomes – Thromboembolic events	(1) 30-day composite of thromboembolic events including stroke, transient ischemic attack, systemic embolism, and myocardial infarction<br>(2) 30-day stroke<br>(3) 30-day transient ischemic attack<br>(4) 30-day systemic embolism<br>(5) 30-day myocardial infarction
Secondary outcomes - Others	(1) 30-day death from any cause<br>(2) 30-day composite of thromboembolic events and death from any cause
Other measurements	(1) Protocol adherence<br>(2) The survey from the operator performing the actual procedure or surgery

Abbreviation: ISTH, the International Society on Thrombosis and Haemostasis.

© 2025 Lee SR et al. *JAMA Network Open*.

eAppendix 5. Definitions of study outcomes

Primary safety outcome

30-day major bleeding event according to the ISTH definition

- Fatal bleeding[†], and/or
- Symptomatic bleeding in a critical area or organ[‡], such as intracranial, intraspinal, intraocular, retroperitoneal, intra-articular or pericardial, or intramuscular with compartment syndrome, and/or
- Bleeding causing a fall in hemoglobin level of 20 g/L (1.24 mmol/L) or more, or leading to transfusion of two or more units of whole blood or red cells.

Secondary outcomes - Bleedings

(1) 30-day clinically relevant nonmajor bleeding (CRNMB)

* Definition of CRNMB (2)

Any sign or symptom of hemorrhage (e.g., more bleeding than would be expected for a clinical circumstance, including bleeding found by imaging alone) that does not fit the criteria for the ISTH definition of major bleeding but does meet at least one of the following criteria:

- Requiring medical intervention by a healthcare professional
- Leading to hospitalization or increased level of care
- Prompting a face-to-face (i.e., not just a telephone or electronic communication) evaluation

(2) 30-day minor bleeding

(3) 30-day all bleeding (a composite of major bleeding, clinically relevant nonmajor bleeding, and minor bleeding)

Secondary outcomes – Thromboembolic events and others

(1) 30-day composite of thromboembolic events, including stroke, transient ischemic attack, systemic embolism, and myocardial infarction
(2) 30-day stroke
(3) 30-day transient ischemic attack
(4) 30-day systemic embolism
(5) 30-day myocardial infarction
(6) 30-day death from any cause
(7) 30-day composite of thromboembolic events and death from any cause

* Definition of systemic embolism

- Ischemic stroke[*]
- Transient ischemic attack[*]
- Acute myocardial infarct
- Deep vein thrombosis
- Pulmonary thromboembolism
- Other venous thromboembolism events

[†] Defined as a hemorrhagic complication leading to death.
[‡] Refers to bleeding that causes specific symptoms requiring immediate medical attention or treatment.
[*] Determined based on the diagnosis of a neurologist or the interpretation findings of a Brain CT/MRI.

© 2025 Lee SR et al. *JAMA Network Open*.

eAppendix 6. Sample size estimation

The following assumptions are made to calculate the number of subjects to meet the study objectives.

- Primary outcome: A 30-day major bleeding event (major bleeding: according to the International Society on Thrombosis and Haemostasis [ISTH] criteria) (1)
- Level of significance, $\alpha = 0.05$
- The power of the test = 80%
- Superiority design based on a single-arm proportional test

The rationale for calculating the number of subjects according to the primary outcome was based on bleeding events. (3-6) The incidence of the primary outcome of discontinuing factor Xa inhibitors for minor bleeding-risk procedures defined by the European Heart Rhythm Association varies across reporters, with dental procedure accounting for 5.6%, 2.5% for cataract surgery, and (2.9%/7.9%) for low-/high-risk gastrointestinal endoscopic procedures. (6,7) Based on the literature, we assumed an average primary outcome rate of 3.6% for all procedures. In contrast, the primary outcome rate when factor Xa inhibitors were maintained was assumed to be 4.8%, based on the existing literature, and because it is conventionally expected to be higher than when factor Xa inhibitors are discontinued. (7) This study aimed to show that the bleeding rate would be lower when factor Xa inhibitors were discontinued than when they were maintained. Moreover, the number of subjects was calculated based on the existing literature, which showed a 4.8% bleeding rate when factor Xa inhibitors were maintained and a 3.6% bleeding rate when they were discontinued. The bleeding rate, when maintained, was set as the null hypothesis (H0: $p = p0$, primary outcome rate of 4.8%), and the bleeding rate, when discontinued, was set as the alternative hypothesis (H1: $p = p1 \neq p0$), primary outcome rate of 3.6%. Based on the above assumptions, the required number of subjects is expected to be 2303, and considering the dropout rate of 8%, the final number of subjects is set to be 2500.

© 2025 Lee SR et al. *JAMA Network Open*.

eAppendix 7. Risk stratification for procedural bleed risk of the procedures included in the PERIXa study from the ISTH and EHRA

ISTH Guidance Statement: Guidance document on the periprocedural management of patients on chronic oral anticoagulant therapy	2021 EHRA Practical Guide on the Use of Non-Vitamin K antagonist Oral Anticoagulants in Patients with Atrial fibrillation
Minimal bleeding risk procedure (30-d risk of major bleed ~0%)[a]	Minor risk interventions (i.e. infrequent bleeding and with low clinical impact)
▪ Ophthalmological (cataract) procedures<br>▪ Minor dental procedures (dental extractions, restorations, prosthetics, endodontics), dental cleanings, fillings	▪ Cataract or glaucoma intervention<br>▪ Dental extraction (1-3 teeth), paradontal surgery, implant positioning, subgingival scaling/cleaning<br>▪ Endoscopy without biopsy or resection
Low/moderate bleeding risk procedures (30-d risk of major bleed 0%-2%)[b]	Low-risk intervention (i.e. infrequent bleeding or with non-severe clinical impact)
▪ Gastrointestinal endoscopy +/- biopsy<br>▪ Colonoscopy +/- biopsy	▪ Complex dental procedures<br>▪ Endoscopy with simple biopsy

[a]Procedure can be safely done under full dose anticoagulation (may consider holding DOAC dose day of procedure to avoid peak anticoagulant effects.)

[b]Some residual anticoagulant effect allowed (ie 2-3 drug half-life interruption preprocedure).

© 2025 Lee SR et al. *JAMA Network Open*.

eTable 1. Procedure characteristics in the modified intention-to-treat analysis set

	Endoscopy (n=948)	Dental procedure (n=820)	Ocular surgery (n=120)	Other procedures (n=14)
**Endoscopy**				
EGD	527 (55.6)	-	-	-
CFS	144 (15.2)	-	-	-
Both EGD & CFS	210 (22.2)	-	-	-
EGD & EMR	1 (0.1)	-	-	-
EGD & ESD	2 (0.2)	-	-	-
CFS & EMR	52 (5.5)	-	-	-
CFS & ESD	7 (0.7)	-	-	-
EGD & CFS & EMR	3 (0.3)	-	-	-
EGD & CFS & ESD	2 (0.2)	-	-	-
**Dental procedure**				
**Dental extraction**	-	505 (61.6)	-	-
**Paradontal procedure**	-	65 (7.9)	-	-
**Incision of abscess**	-	7 (0.9)	-	-
**Implant**	-	152 (18.5)	-	-
Dental extraction & paradontal procedure	-	6 (0.7)	-	-
Dental extraction & I&D	-	1 (0.1)	-	-
Dental extraction & implant	-	75 (9.1)	-	-

© 2025 Lee SR et al. *JAMA Network Open*.

Paradontal procedure & implant	-	3 (0.4)	-	-
Dental extraction & paradontal procedure & implant	-	4 (0.5)	-	-
Dental extraction & paradontal & I&D & implant	-	2 (0.2)	-	-
**Ocular surgery**				
Cataract surgery	-	-	116 (96.7)	-
Glaucoma surgery	-	-	4 (3.3)	-
**Other procedure**				
Both dental & endoscopy	-	-	-	5 (35.7)
Pterygium Surgery	-	-	-	1 (7.1)
Face lift surgery	-	-	-	1 (7.1)
lower blepharoplasty	-	-	-	1 (7.1)
Correction of Blepharoptosis	-	-	-	1 (7.1)
Vitrectomy	-	-	-	2 (14.3)
Eye procedure	-	-	-	1 (7.1)
Percutaneous Epidural Neuroplasty	-	-	-	1 (7.1)
Thyroid biopsy	-	-	-	1 (7.1)

Numbers are mean (standard deviation) or n (%).

Abbreviations: CFS, colonofibroscopy; EGD, esophagogastroduodenoscopy; EMR, endoscopic mucosal resection; ESD, endoscopic submucosal dissection; I&D, incision and drainage.

© 2025 Lee SR et al. *JAMA Network Open*.

eTable 2. Baseline characteristics according to the DOAC types and DOAC regimens in the modified intention-to-treat analysis set

	Apixaban (n=921)	Edoxaban (n=616)	Rivaroxaban (n=365)	P-value	Twice daily (n=921)	Once daily (n=981)	P-value
Demographics							
**Age, median (IQR)**	70.0 (65.0-76.0)	72.0 (66.0-77.0)	70.0 (65.0-76.0)	.06	70.0 (65.0-76.0)	71.0 (65.0-76.0)	.97
**<65 years**	200 (21.7)	121 (19.6)	90 (24.7)	.14	200 (21.7)	211 (21.5)	.51
**65-74 years**	436 (47.3)	275 (44.6)	168 (46.0)		436 (47.3)	443 (45.2)	
**≥75 years**	285 (30.9)	220 (35.7)	107 (29.3)		285 (30.9)	327 (33.3)	
**Female, %**	389 (42.2)	254 (41.2)	124 (34.0)	.02	389 (42.2)	378 (38.5)	.11
**CHA_2DS_2-VASc, median (IQR)**	3.0 (2.0-4.0)	3.0 (2.0-4.0)	3.0 (2.0-4.0)	.12	3.0 (2.0-4.0)	3.0 (2.0-4.0)	.40
**CHA_2DS_2-VASc < 3**	422 (46.0)	247 (40.7)	164 (45.3)		422 (46.0)	411 (42.4)	.13
**CHA_2DS_2-VASc ≥ 3**	495 (54.0)	360 (59.3)	198 (54.7)		495 (54.0)	558 (57.6)	
**HAS-BLED, median (IQR)**	2.0 (1.0-2.0)	2.0 (1.0-2.0)	2.0 (1.0-2.0)	.37	2.0 (1.0-2.0)	2.0 (1.0-2.0)	.17
**HAS-BLED < 3**	839 (91.6)	553 (91.4)	327 (90.1)		839 (91.6)	880 (90.9)	.66
**HAS-BLED ≥ 3**	77 (8.4)	52 (8.6)	36 (9.9)		77 (8.4)	88 (9.1)	
Comorbidities							
**Hypertension**	612 (66.4)	428 (69.5)	260 (71.2)	.19	612 (66.4)	688 (70.1)	.09
**Diabetes mellitus**	238 (25.8)	157 (25.5)	104 (28.5)	.55	238 (25.8)	261 (26.6)	.74

© 2025 Lee SR et al. *JAMA Network Open*.

**CHF**	126 (13.7)	86 (14.0)	45 (12.3)	.75	126 (13.7)	131 (13.4)	.89
**CKD**	56 (6.1)	30 (4.9)	20 (5.5)	.60	56 (6.1)	50 (5.1)	.40
**Dialysis**	6 (0.7)	2 (0.3)	1 (0.3)	.54	6 (0.7)	3 (0.3)	.45
**Chronic liver disease**	13 (1.4)	6 (1.0)	9 (2.5)	.17	13 (1.4)	15 (1.5)	.98
**Stroke/TIA/TE**	78 (8.5)	56 (9.1)	28 (7.7)	.74	78 (8.5)	84 (8.6)	1
**Previous DCC**	177 (19.2)	95 (15.4)	57 (15.6)	.10	177 (19.2)	152 (15.5)	.04
**Previous RFCA**	212 (23.0)	124 (20.1)	81 (22.2)	.40	212 (23.0)	205 (20.9)	.29
**Previous ACS**	17 (1.8)	13 (2.1)	15 (4.1)	.05	17 (1.8)	28 (2.9)	.20
**Previous PCI**	32 (3.5)	30 (4.9)	20 (5.5)	.20	32 (3.5)	50 (5.1)	.10
Medications							
**Apixaban**				<.001			<.001
**5 mg twice daily**	679 (73.7)	-	-		679 (73.7)	0 (0.0)	
**2.5 mg twice daily**	242 (26.3)	-	-		242 (26.3)	0 (0.0)	
**Edoxaban**				<.001			<.001
**60 mg once daily**	-	312 (50.6)	-		0 (0.0)	312 (31.8)	
**30 mg once daily**	-	292 (47.4)	-		0 (0.0)	292 (29.8)	
**15 mg once daily**	-	12 (1.9)	-		0 (0.0)	12 (1.2)	
**Rivaroxaban**				<.001	0 (0.0)	365 (37.2)	<.001
**20 mg once daily**	-	-	136 (37.3)		0 (0.0)	136 (13.9)	
**15 mg once daily**	-	-	222 (60.8)		0 (0.0)	222 (22.6)	
**10 mg once daily**	-	-	7 (1.9)		0 (0.0)	7 (0.7)	
**Class Ic AAD**	390 (42.3)	166 (26.9)	127 (34.8)	<.001	390 (42.3)	293 (29.9)	<.001

© 2025 Lee SR et al. *JAMA Network Open*.

**Class III AAD**	149 (16.2)	107 (17.4)	60 (16.4)	.82	149 (16.2)	167 (17.0)	.67
**Beta blockers**	471 (51.1)	286 (46.4)	214 (58.6)	.001	471 (51.1)	500 (51.0)	.98
**CCB**	581 (63.1)	430 (69.8)	243 (66.6)	.02	581 (63.1)	673 (68.6)	.01
**Digoxin**	54 (5.9)	18 (2.9)	22 (6.0)	.02	54 (5.9)	40 (4.1)	.09
**ACEi**	22 (2.4)	17 (2.8)	9 (2.5)	.90	22 (2.4)	26 (2.7)	.83
**ARB**	329 (35.7)	277 (45.0)	139 (38.1)	.001	329 (35.7)	416 (42.4)	.003
**Diuretics**	216 (23.5)	130 (21.1)	78 (21.4)	.50	216 (23.5)	208 (21.2)	.26
**Statin**	421 (45.7)	308 (50.0)	188 (51.5)	.10	421 (45.7)	496 (50.6)	.04
**NSAID**	4 (0.4)	5 (0.8)	1 (0.3)	.46	4 (0.4)	6 (0.6)	.83
**PPI**	149 (16.2)	96 (15.6)	48 (13.2)	.39	149 (16.2)	144 (14.7)	.40
**H2-blockers**	17 (1.8)	19 (3.1)	5 (1.4)	.14	17 (1.8)	24 (2.4)	.46
Lab findings							
**Platelet, median (IQR), x10^3/uL**	198.5 (166.0-241.00	205.0 (172.0-240.0)	201.0 (169.0-234.0)	.75	198.5 (166.0-241.0)	203.0 (170.0-237.0)	.71
**PT, median (IQR), INR**	1.1 (1.0-1.2)	1.1 (1.0-1.2)	1.2 (1.1-1.4)	.003	1.1 (1.0-1.2)	1.1 (1.0-1.3)	.21
**aPTT, median (IQR), sec**	31.9 (28.4-36.1)	32.5 (29.4-37.0)	35.4 (31.1-39.7)	<.001	31.9 (28.4-36.1)	33.6 (30.3-38.0)	.001
**Creatinine, median (IQR), mg/dL**	0.9 (0.8-1.0)	0.9 (0.8-1.0)	0.9 (0.8-1.1)	.07	0.9 (0.8-1.0)	0.9 (0.8-1.0)	.84
**eGFR, MDRD, median (IQR), min/ml/1.73m^2**	75.5 (65.0-86.3)	77.2 (65.7-87.3)	74.8 (63.1-87.5)	.37	75.5 (65.0-86.3)	76.4 (64.8-87.5)	.31
**AST, median (IQR), IU/L**	23.0 (19.0-28.0)	23.0 (20.0-30.0)	24.0 (20.0-29.5)	.29	23.0 (19.0-28.0)	23.0 (20.0-30.0)	.11

© 2025 Lee SR et al. *JAMA Network Open*.

**ALT, median (IQR), IU/L**	19.0 (14.0-26.0)	19.0 (15.0-27.0)	19.0 (15.0-26.0)	.41	19.0 (14.0-26.0)	19.0 (15.0-27.0)	.19
**LA volume, median (IQR), mL**	62.4 (47.0-92.0)	62.0 (47.0-78.0)	71.2 (50.5-104.0)	.09	62.4 (47.0-92.0)	65.0 (48.7-90.0)	.72
**LA volume index, median (IQR), mL/m^2**	48.6 (37.1-65.2)	42.4 (34.0-54.7)	43.6 (36.3-57.2)	.08	48.6 (37.1-65.2)	42.7 (34.0-56.2)	.04
**LV ejection fraction, median (IQR), %**	59.0 (56.0-63.3)	60.0 (56.0-64.0)	60.3 (56.0-64.5)	.75	59.0 (56.0-63.3)	60.0 (56.0-64.0)	.52
**BMI, median (IQR), kg/m2**	24.5 (22.5-26.8)	24.7 (22.7-26.7)	25.3 (23.1-27.7)	.003	24.5 (22.5-26.8)	24.9 (22.8-27.0)	.03
**Smoking**				<.001			<.001
**- Never**	440 (47.8)	265 (43.0)	125 (34.2)		440 (47.8)	390 (39.8)	
**- Former (quit > 2 months ago)**	420 (45.6)	283 (45.9)	186 (51.0)		420 (45.6)	469 (47.8)	
**- Current**	29 (3.1)	43 (7.0)	32 (8.8)		29 (3.1)	75 (7.6)	
**- Unknown**	32 (3.5)	25 (4.1)	22 (6.0)		32 (3.5)	47 (4.8)	
**Alcohol**				<.001			.001
**- No**	394 (42.8)	262 (42.5)	171 (46.8)		394 (42.8)	433 (44.1)	
**- Social**	42 (4.6)	47 (7.6)	25 (6.8)		42 (4.6)	72 (7.3)	
**- Yes**	75 (8.1)	58 (9.4)	50 (13.7)		75 (8.1)	108 (11.0)	
**- Unknown**	410 (44.5)	249 (40.4)	119 (32.6)		410 (44.5)	368 (37.5)	

Numbers are mean (standard deviation) or n (%).

© 2025 Lee SR et al. *JAMA Network Open*.

Abbreviations: ACEi, angiotensin converting enzyme inhibitor; ACS, acute coronary syndrome; AF, atrial fibrillation; aPTT, activated partial thromboplastin time; ARB, angiotensin receptor blocker; BMI, body mass index; CCB, calcium channel blocker; CHF, congestive heart failure; CKD, chronic kidney disease; DCC, direct current cardioversion; eGFR, estimated glomerular filtration rate; INR, international normalized ratio; LA, left atrium; LV, left ventricle; MDRD, Modification of Diet in Renal Disease; NSAID, non-steroidal anti-inflammatory drug; PCI, percutaneous coronary intervention; PPI, proton-pump inhibitor; PT, prothrombin time; RFCA, radiofrequency catheter ablation; TE, thromboembolism; TIA, transient ischemic attack.

SI conversion factors: To convert platelet count x10^3/uL to x10^9/L, multiply by 1; Creatinine to micromole per liter, multiply 88.4; AST and ALT to microkatals per liter, multiply by 0.0167.

© 2025 Lee SR et al. *JAMA Network Open*.

eTable 3. Clinical outcomes at each visit according to the procedures in modified intention-to-treat analysis set

	Overall (N=1902)	Endoscopy (n=948)	Dental procedure (n=820)	Ocular surgery (n=120)	Other procedures (n=14)	P-value
Procedure day						
Major bleeding	0	0	0	0	0	-
CRNMB	5 (0.3)	0	5 (0.6)	0	0	.09
Minor bleeding	22 (1.2)	3 (0.3)	17 (2.1)	0	2 (15.4)	<.001
Stroke	0	0	0	0	0	-
Transient ischemic attack	0	0	0	0	0	-
Systemic embolism	0	0	0	0	0	-
Myocardial infarction	0	0	0	0	0	-
Death from any cause	0	0	0	0	0	-
Visit 1						
Major bleeding	1 (0.1)	0	1 (0.1)	0	0	.72
CRNMB	4 (0.2)	1 (0.1)	3 (0.4)	0	0	.63
Minor bleeding	22 (1.2)	4 (0.4)	18 (2.2)	0	0	.003
Stroke	0	0	0	0	0	-
Transient ischemic attack	0	0	0	0	0	-
Systemic embolism	0	0	0	0	0	-
Myocardial infarction	0	0	0	0	0	-
Death from any cause	0	0	0	0	0	-
Visit 2						
Major bleeding	1 (0.1)	0	1 (0.1)	0	0	.72
CRNMB	0	0	0	0	0	-
Minor bleeding	5 (0.3)	1 (0.1)	4 (0.5)	0	0	.42
Stroke	0	0	0	0	0	-

© 2025 Lee SR et al. *JAMA Network Open*.

Transient ischemic attack	0	0	0	0	0	-
Systemic embolism	0	0	0	0	0	-
Myocardial infarction	0	0	0	0	0	-
Death from any cause	0	0	0	0	0	-

Numbers are mean (standard deviation) or n (%).

© 2025 Lee SR et al. *JAMA Network Open*.

eTable 4. Post-procedural questionnaire from the operators according to the DOAC types in the modified intention-to-treat analysis set

	Apixaban (n=921)	Edoxaban (n=616)	Rivaroxaban (n=365)	P-value
**Survey response rate**	541 (58.7)	370 (60.1)	247 (67.7)	
**(A) None at all**	115 (21.3)	92 (24.9)	53 (21.5)	.52
**(B) Similar to the expected level in a general patient not on anticoagulation**	360 (66.5)	243 (65.7)	157 (63.6)	
**(C) More bleeding during or immediately after the procedure than in a general patient not on anticoagulation, but controllable during the procedure**	62 (11.5)	34 (9.2)	37 (15.0)	
**(D) More bleeding during or immediately after the procedure than in a general patient not on anticoagulation, requiring special measures for hemostasis**	2 (0.4)	1 (0.3)	0 (0.0)	
**(E) Post-procedure/surgery re-intervention or additional endoscopy due to delayed bleeding**	1 (0.2)	0 (0.0)	0 (0.0)	
**(F) Other**	1 (0.2)	0 (0.0)	0 (0.0)	

Numbers are n (%).

The percentages in the table were calculated based on the number of respondents.

© 2025 Lee SR et al. *JAMA Network Open*.

eTable 5. Post-procedural questionnaire from the operators according to the DOAC regimens in the modified intention-to-treat analysis set

	Twice daily (n=921)	Once daily (n=981)	P-value
**Survey response rate**	541 (58.7)	617 (62.9)	
**(A) None at all**	115 (21.3)	145 (23.5)	.62
**(B) Similar to the expected level in a general patient not on anticoagulation**	360 (66.5)	400 (64.8)	
**(C) More bleeding during or immediately after the procedure than in a general patient not on anticoagulation, but controllable during the procedure**	62 (11.5)	71 (11.5)	
**(D) More bleeding during or immediately after the procedure than in a general patient not on anticoagulation, requiring special measures for hemostasis**	2 (0.4)	1 (0.2)	
**(E) Post-procedure/surgery re-intervention or additional endoscopy due to delayed bleeding**	1 (0.2)	0 (0.0)	
**(F) Other**	1 (0.2)	0 (0.0)	

Numbers are n (%).

The percentages in the table were calculated based on the number of respondents.

© 2025 Lee SR et al. *JAMA Network Open*.

eTable 6. The reason for protocol violation in the modified intention-to-treat analysis set

		Number (%)*	Endoscopy	Dental procedure	Ocular surgery	Other procedure
Pre	Early omission	166 (8.7)	93 (4.9)	58 (3.0)	13 (0.7)	2 (0.1)
	Delayed omission	10 (0.5)	6 (0.3)	4 (0.2)	0 (0.0)	0 (0.0)
	**P-value**	.80				
Pre & post	Early omission & resumption	15 (0.8)	7 (0.4)	7 (0.4)	1 (0.1)	0 (0.0)
	Delayed omission & early resumption	12 (0.6)	6 (0.3)	4 (0.2)	2 (0.1)	0 (0.0)
	Early omission & delayed resumption	9 (0.5)	3 (0.2)	4 (0.2)	1 (0.1)	1 (0.1)
	Delayed omission & resumption	1 (0.1)	0 (0.0)	1 (0.1)	0 (0.0)	0 (0.0)
	Continued during procedure, delayed omission 1day after procedure, and resumption 2days later	1 (0.1)	0 (0.0)	1 (0.1)	0 (0.0)	0 (0.0)
	**P-value**	.85				
Post	Early resumption	25 (1.3)	7 (0.4)	16 (0.8)	2 (0.1)	0 (0.0)
	Delayed resumption	23 (1.2)	8 (0.4)	13 (0.7)	2 (0.1)	0 (0.0)
	**P-value**	1.000				

Numbers are n (%).

*** The number (%) was calculated using the total number of patients (N=1902) as the denominator.**

© 2025 Lee SR et al. *JAMA Network Open*.

eTable 7. Baseline characteristics of participants in the per-protocol analysis set

	Overall (N=1615)	Endoscopy (n=817)	Dental procedure (n=702)	Ocular surgery (n=96)	P-value
Demographics					
**Age, median (IQR), year**	71.0 (65.0-76.0)	69.0 (64.0-75.0)	72.0 (66.0-78.0)	74.5 (70.0-80.5)	<.001
**<65 years**	347 (21.5)	205 (25.1)	136 (19.4)	6 (6.2)	
**65-74 years**	755 (46.7)	401 (49.1)	312 (44.4)	42 (43.8)	
**≥75 years**	513 (31.8)	211 (25.8)	254 (36.2)	48 (50.0)	
**Female, %**	655 (40.6)	318 (38.9)	289 (41.2)	48 (50.0)	.10
**CHA_2DS_2-VASc, median (IQR)**	3.0 (2.0-4.0)	2.0 (2.0-3.0)	3.0 (2.0-4.0)	3.0 (3.0-4.0)	<.001
**CHA_2DS_2-VASc < 3**	697 (43.5)	408 (50.2)	271 (39.1)	18 (18.8)	<.001
**CHA_2DS_2-VASc ≥ 3**	905 (56.5)	405 (49.8)	422 (60.9)	78 (81.2)	
**HAS-BLED, median (IQR)**	3.0 (2.0-3.0)	3.0 (2.0-3.0)	3.0 (2.0-3.0)	3.0 (2.0-3.0)	.002
**HAS-BLED < 3**	1453 (90.8)	739 (91.1)	623 (89.8)	91 (94.8)	.25
**HAS-BLED ≥ 3**	148 (9.2)	72 (8.9)	71 (10.2)	5 (5.2)	
Comorbidities					
**Hypertension**	1124 (69.6)	561 (68.7)	490 (69.8)	73 (76.0)	.33
**Diabetes mellitus**	428 (26.5)	193 (23.6)	194 (27.6)	41 (42.7)	<.001
**CHF**	219 (13.6)	97 (11.9)	104 (14.8)	18 (18.8)	.08
**CKD**	94 (5.8)	38 (4.7)	46 (6.6)	10 (10.4)	.04

© 2025 Lee SR et al. *JAMA Network Open*.

**Dialysis**	7 (0.4)	2 (0.2)	5 (0.7)	0 (0.0)	.31
**Chronic liver disease**	23 (1.4)	11 (1.3)	10 (1.4)	2 (2.1)	.85
**Stroke/TIA/TE**	143 (8.9)	60 (7.3)	78 (11.1)	5 (5.2)	.02
**Previous DCC**	283 (17.5)	154 (18.8)	117 (16.7)	12 (12.5)	.22
**Previous RFCA**	362 (22.4)	219 (26.8)	131 (18.7)	12 (12.5)	<.001
**Previous ACS**	39 (2.4)	12 (1.5)	23 (3.3)	4 (4.2)	.04
**Previous PCI**	76 (4.7)	28 (3.4)	44 (6.3)	4 (4.2)	.03
Medications					
**Apixaban**	805 (49.8)	424 (51.9)	342 (48.7)	39 (40.6)	.08
**5 mg twice daily**	598 (37.0)	334 (40.9)	238 (33.9)	26 (27.1)	.006
**2.5 mg twice daily**	207 (12.8)	90 (11.0)	104 (14.8)	13 (13.5)	
**Edoxaban**	509 (31.5)	263 (32.2)	214 (30.5)	32 (33.3)	.72
**60 mg once daily**	253 (15.7)	138 (16.9)	104 (14.8)	11 (11.5)	.12
**30 mg once daily**	247 (15.3)	124 (15.2)	103 (14.7)	20 (20.8)	
**15 mg once daily**	9 (0.6)	1 (0.1)	7 (1.0)	1 (1.0)	
**Rivaroxaban**	301 (18.6)	130 (15.9)	146 (20.8)	25 (26.0)	.008
**20 mg once daily**	119 (7.4)	52 (6.4)	60 (8.5)	7 (7.3)	.007
**15 mg once daily**	177 (11.0)	78 (9.5)	81 (11.5)	18 (18.8)	
**10 mg once daily**	5 (0.3)	0 (0.0)	5 (0.7)	0 (0.0)	
**Class Ic AAD**	591 (36.6)	320 (39.2)	248 (35.3)	23 (24.0)	.009
**Class III AAD**	276 (17.1)	156 (19.1)	107 (15.2)	13 (13.5)	.09
**Beta blockers**	842 (52.1)	423 (51.8)	366 (52.1)	53 (55.2)	.82

© 2025 Lee SR et al. *JAMA Network Open*.

**CCB**	1083 (67.1)	537 (65.7)	484 (68.9)	62 (64.6)	.36
**Digoxin**	82 (5.1)	33 (4.0)	43 (6.1)	6 (6.2)	.16
**ACEi**	42 (2.6)	17 (2.1)	22 (3.1)	3 (3.1)	.41
**ARB**	652 (40.4)	323 (39.5)	289 (41.2)	40 (41.7)	.78
**Diuretics**	363 (22.5)	163 (20.0)	169 (24.1)	31 (32.3)	.009
**Statin**	789 (48.9)	398 (48.7)	352 (50.1)	39 (40.6)	.22
**NSAID**	7 (0.4)	3 (0.4)	3 (0.4)	1 (1.0)	.64
**PPI**	259 (16.0)	156 (19.1)	95 (13.5)	8 (8.3)	.001
**H2-blockers**	38 (2.4)	23 (2.8)	14 (2.0)	1 (1.0)	.39
Lab findings					
**Platelet, median (IQR), x10^3/uL**	201.0 (169.0-240.0)	204.0 (170.0-241.0)	200.0 (167.0-235.0)	196.0 (157.0-250.5)	.44
**PT, median (IQR), INR**	1.1 (1.0-1.2)	1.1 (1.0-1.2)	1.1 (1.0-1.2)	1.2 (1.1-1.4)	.10
**aPTT, median (IQR), sec**	32.9 (28.9-37.0)	31.9 (29.1-36.8)	33.8 (28.2-37.5)	34.2 (30.9-37.9)	.17
**Creatinine, median (IQR), mg/dL**	0.9 (0.8-1.0)	0.9 (0.8-1.0)	0.9 (0.8-1.1)	0.9 (0.8-1.1)	.045
**eGFR, MDRD, median (IQR), min/ml/1.73m^2**	75.6 (64.0-87.0)	77.7 (66.4-88.6)	74.3 (62.4-85.7)	68.9 (54.1-82.7)	<0.001
**AST, median (IQR), IU/L**	23.0 (20.0-29.0)	24.0 (20.0-29.0)	23.0 (19.0-29.5)	21.0 (19.0-25.0)	0.01
**ALT, median (IQR), IU/L**	19.0 (14.0-27.0)	20.0 (15.0-27.0)	19.0 (14.0-27.0)	16.0 (12.0-21.0)	0.001
**LA volume, median (IQR), mL**	63.9 (47.6-90.0)	64.0 (48.0-91.0)	62.9 (47.0-87.0)	64.0 (54.0-119.9)	.53
**LA volume index, median (IQR), mL/m^2**	44.0 (34.9-58.0)	44.2 (36.4-57.0)	43.0 (33.6-59.1)	49.4 (39.5-69.3)	.34
**LV ejection fraction, median (IQR), %**	60.0 (56.0-64.0)	60.0 (56.0-65.0)	59.0 (56.0-63.7)	60.0 (56.2-63.9)	.42
**BMI, median (IQR), kg/m2**	24.8 (22.7-26.9)	24.6 (22.6-26.6)	25.0 (22.7-27.4)	24.8 (22.7-27.7)	.19

© 2025 Lee SR et al. *JAMA Network Open*.

**Smoking**					.02
**- Never**	773 (47.9)	362 (44.3)	365 (52.0)	46 (47.9)	
**- Former (quit > 2 months ago)**	85 (5.3)	40 (4.9)	42 (6.0)	3 (3.1)	
**- Current**	66 (4.1)	34 (4.2)	30 (4.3)	2 (2.1)	
**- Unknown**	691 (42.8)	381 (46.6)	265 (37.7)	45 (46.9)	
**Alcohol**					.02
**- No**	718 (44.5)	341 (41.7)	332 (47.3)	45 (46.9)	
**- Social**	105 (6.5)	46 (5.6)	56 (8.0)	3 (3.1)	
**- Yes**	144 (8.9)	72 (8.8)	66 (9.4)	6 (6.2)	
**- Unknown**	648 (40.1)	358 (43.8)	248 (35.3)	42 (43.8)	

Numbers are mean (standard deviation) or n (%).

Abbreviations: AAD, antiarrhythmic drug; ACEi, angiotensin converting enzyme inhibitor; ACS, acute coronary syndrome; AF, atrial fibrillation; aPTT, activated partial thromboplastin time; ARB, angiotensin receptor blocker; BMI, body mass index; CCB, calcium channel blocker; CHF, congestive heart failure; CKD, chronic kidney disease; DCC, direct current cardioversion; eGFR, estimated glomerular filtration rate; INR, international normalized ratio; LA, left atrium; LV, left ventricle; MDRD, Modification of Diet in Renal Disease; NSAID, non-steroidal anti-inflammatory drug; PCI, percutaneous coronary intervention; PPI, proton-pump inhibitor; PT, prothrombin time; RFCA, radiofrequency catheter ablation; TE, thromboembolism; TIA, transient ischemic attack.

SI conversion factors: To convert platelet count $x10^3$/uL to $x10^9$/L, multiply by 1; Creatinine to micromole per liter, multiply 88.4; AST and ALT to microkatals per liter, multiply by 0.0167.

© 2025 Lee SR et al. *JAMA Network Open*.

eTable 8. Clinical outcomes of participants who followed protocol according to the procedure in the per-protocol analysis set

	Overall<br><br>(N=1615)	Endoscopy<br><br>(n=817)	Dental procedure<br><br>(n=702)	Ocular surgery<br><br>(n=96)	P-value
**Primary outcome**					
**Major bleeding**	2 (0.1)	0	2 (0.3)	0	.27
**Secondary outcomes**					
**Composite of thromboembolic events***	0	0	0	0	-
**Bleedings**	0	0	0	0	-
CRNMB	5 (0.3)	1 (0.1)	4 (0.6)	0 (0.0)	.25
Minor bleeding	29 (1.8)	6 (0.7)	23 (3.3)	0 (0.0)	<.001
All bleeding	33 (2.0)	6 (0.7)	27 (3.8)	0	<.001
**Thromboembolic events**					
Stroke	0	0	0	0	-
Transient ischemic attack	0	0	0	0	-
Systemic embolism	0	0	0	0	-
Myocardial infarction	0	0	0	0	-
**Others**					
Death from any cause	0	0	0	0	-
Composite of thromboembolic events and all-cause death	0	0	0	0	-

Procedure day

© 2025 Lee SR et al. *JAMA Network Open*.

Major bleeding	0	0	0	0	-
CRNMB	2 (0.1)	0	2 (0.3)	0	.27
Minor bleeding	13 (0.8)	2 (0.2)	11 (1.6)	0	.01
Stroke	0	0	0	0	-
Transient ischemic attack	0	0	0	0	-
Systemic embolism	0	0	0	0	-
Myocardial infarction	0	0	0	0	-
Death from any cause	0	0	0	0	-
Visit 1					
Major bleeding	1 (0.1)	0	1 (0.1)	0	.52
CRNMB	4 (0.2)	1 (0.1)	3 (0.4	0	.43
Minor bleeding	16 (1.0)	3 (0.4)	13 (1.9)	0	.009
Stroke	0	0	0	0	-
Transient ischemic attack	0	0	0	0	-
Systemic embolism	0	0	0	0	-
Myocardial infarction	0	0	0	0	-
Death from any cause	0	0	0	0	-
Visit 2					
Major bleeding	1 (0.1)	0	1 (0.1)	0	.52
CRNMB	0	0	0	0	-
Minor bleeding	4 (0.2)	1 (0.1)	3 (0.4)	0	.43
Stroke	0	0	0	0	-

© 2025 Lee SR et al. *JAMA Network Open*.

Transient ischemic attack	0	0	0	0	-
Systemic embolism	0	0	0	0	-
Myocardial infarction	0	0	0	0	-
Death from any cause	0	0	0	0	-

Numbers are n (%).

* Including stroke, transient ischemic attack, systemic embolism, and myocardial infarction.

Abbreviations: CRNMB, clinically relevant non major bleeding.

© 2025 Lee SR et al. *JAMA Network Open*.

eTable 9. Post-procedural questionnaire from the operators for participants in the per-protocol analysis set

	Overall (N=1615)	Endoscopy (N=817)	Dental procedure (N=702)	Ocular surgery (N=96)
**Survey response rate**	1023 (63.3)	490 (60.0)	475 (67.7)	58 (60.4)
**(A) None at all**	226 (22.1)	167 (34.1)	44 (9.3)	15 (25.9)
**(B) Similar to the expected level in a general patient not on anticoagulation**	683 (66.8)	299 (61.0)	343 (72.2)	41 (70.7)
**(C) More bleeding during or immediately after the procedure than in a general patient not on anticoagulation, but controllable during the procedure**	109 (10.7)	23 (4.7)	84 (17.7)	2 (3.4)
**(D) More bleeding during or immediately after the procedure than in a general patient not on anticoagulation, requiring special measures for hemostasis**	3 (0.3)	0 (0.0)	3 (0.6)	0 (0.0)
**(E) Post-procedure/surgery re-intervention or additional endoscopy due to delayed bleeding**	1 (0.1)	1 (0.2)	0 (0.0)	0 (0.0)
**(F) Other**	1 (0.1)	0 (0.0)	1 (0.2)	0 (0.0)

Numbers are n (%).

The percentages in the table were calculated based on the number of respondents.

© 2025 Lee SR et al. *JAMA Network Open*.

eFigure 1. Perioperative direct oral anticoagulant management protocol for minimal to low bleed risk intervention and PERIXa study design

eFigure 1A

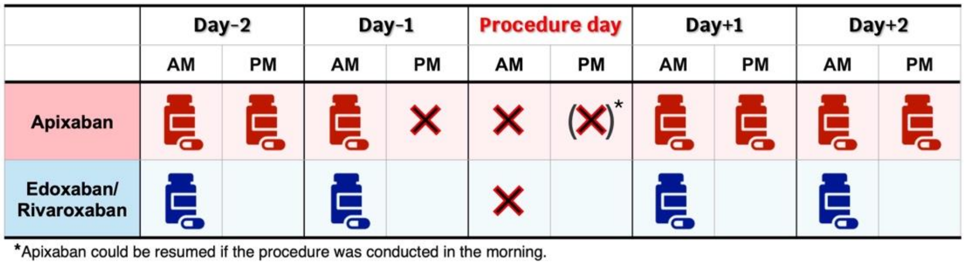

eFigure 1B

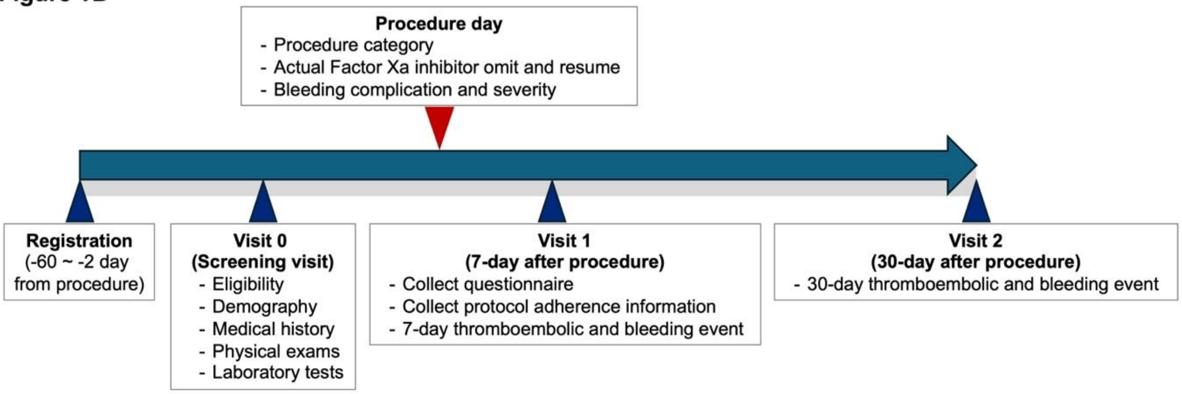

(A) The protocol for discontinuing and resuming direct factor Xa inhibitors in the PERIXa protocol
(B) The flow diagram of the study
Abbreviation: AM, morning; PM, afternoon

© 2025 Lee SR et al. *JAMA Network Open*.

eFigure 2. Procedure characteristics

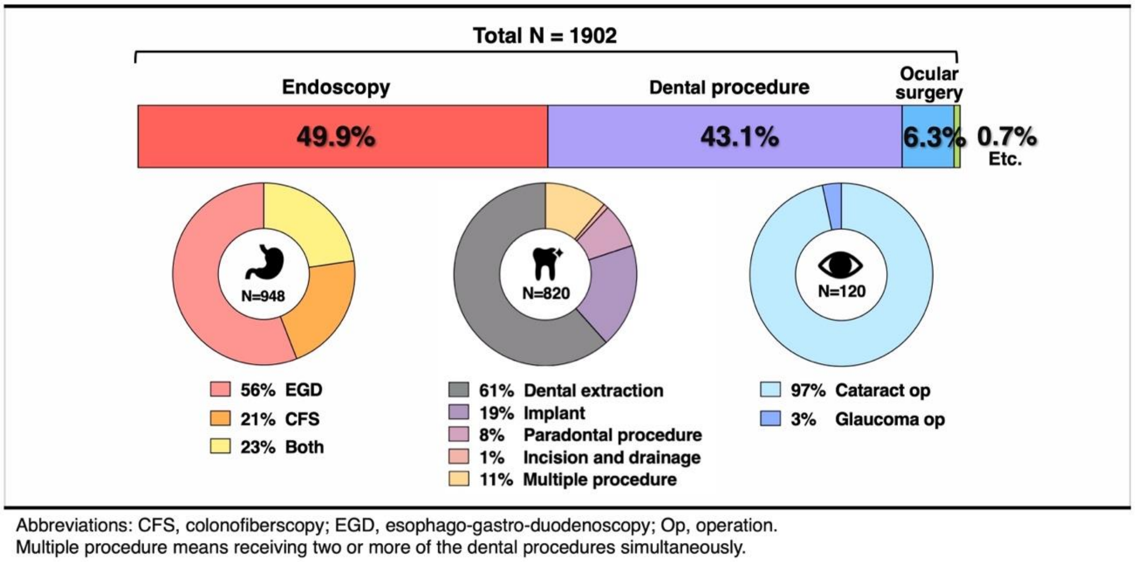

Abbreviation: CFS, colonofibroscopy; EGD, esophagogastroduodenoscopy

© 2025 Lee SR et al. *JAMA Network Open*.

eFigure 3. Central Illustration: a simplified protocol for the discontinuation and resumption of factor Xa inhibitor periprocedurally in patients with atrial fibrillation undergoing minimal to low bleed risk procedures

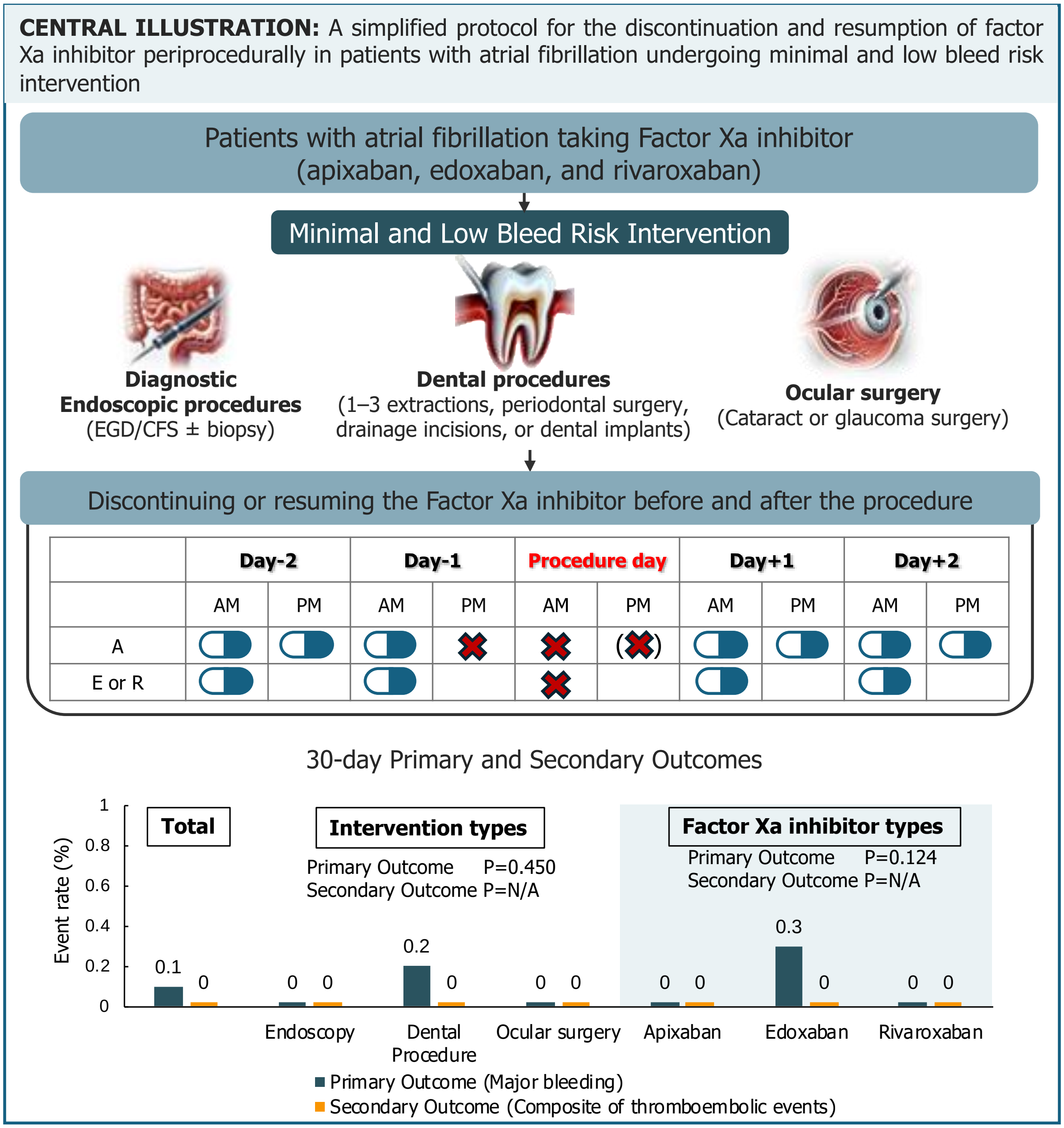

Abbreviation: A, apixaban; AM, morning; CFS, colonofibroscopy; E, edoxaban; EGD, esophagogastroduodenoscopy; N/A, Not Applicable; PM, afternoon; R, rivaroxaban.

© 2025 Lee SR et al. *JAMA Network Open*.

eReferences.

1. Schulman S, Kearon C. Definition of major bleeding in clinical investigations of antihemostatic medicinal products in non-surgical patients. J Thromb Haemost 2005;3:692-4.
2. Kaatz S, Ahmad D, Spyropoulos AC, Schulman S. Definition of clinically relevant non-major bleeding in studies of anticoagulants in atrial fibrillation and venous thromboembolic disease in non-surgical patients: communication from the SSC of the ISTH. J Thromb Haemost 2015;13:2119-26.
3. Douketis JD, Spyropoulos AC, Duncan J et al. Perioperative Management of Patients With Atrial Fibrillation Receiving a Direct Oral Anticoagulant. JAMA Intern Med 2019;179:1469-1478.
4. Sacco R, Sacco M, Carpenedo M, Mannucci PM. Oral surgery in patients on oral anticoagulant therapy: a randomized comparison of different intensity targets. Oral Surg Oral Med Oral Pathol Oral Radiol Endod 2007;104:e18-21.
5. Cheung JJC, Liu S, Li KKW. Phacoemulsification cataract surgery in patients receiving novel oral anticoagulant medications. Int Ophthalmol 2019;39:623-630.
6. Yasuda R, Yoshida N, Murakami T et al. Multicenter Study of the Hemorrhage Risk after Endoscopic Mucosal Resection Associated with Direct Oral Anticoagulants. Gastroenterol Res Pract 2019;2019:5743561.
7. Beyer-Westendorf J, Gelbricht V, Förster K et al. Peri-interventional management of novel oral anticoagulants in daily care: results from the prospective Dresden NOAC registry. Eur Heart J 2014;35:1888-96.

© 2025 Lee SR et al. *JAMA Network Open*.
