# Supplementary material for: Perioperative Factor Xa Inhibitor Discontinuation for Patients Undergoing Procedures With Minimal or Low Bleeding Risk
Source: JAMA Netw Open. 2025 Feb 7;8(2):e2458742. doi: 10.1001/jamanetworkopen.2024.58742 (PMC11806392; doi:10.1001/jamanetworkopen.2024.58742)
Supplement: Supplement 3. — Data Sharing Statement [file jamanetwopen-e2458742-s003.pdf]

Data Sharing Statement

Lee. Perioperative Factor Xa Inhibitor Discontinuation for Patients Undergoing Procedures With Minimal or Low Bleeding Risk. *JAMA Netw Open*. Published February 07, 2025. doi:10.1001/jamanetworkopen.2024.58742

Data
Data available: No

Additional Information
Explanation for why data not available: EKC had full access to all the data in the study and takes responsibility for the integrity of the data and the accuracy of the data analysis. We will permit limited data sharing upon appropriate data access requests. De-identified individual participant data will be made available to investigators whose proposed use has been approved by the trial leadership committee. For any inquiries regarding data sharing, please contact the corresponding author via email.
